# The effect of spermidine on autoimmunity and beta cell function in NOD mice

**DOI:** 10.1038/s41598-022-08168-2

**Published:** 2022-03-16

**Authors:** Ceren Karacay, Barbara Prietl, Clemens Harer, Barbara Ehall, Christoph W. Haudum, Kaddour Bounab, Joakim Franz, Tobias Eisenberg, Frank Madeo, Dagmar Kolb, Kerstin Hingerl, Markus Hausl, Christoph Magnes, Selma I. Mautner, Petra Kotzbeck, Thomas R. Pieber

**Affiliations:** 1 Division of Endocrinology and Diabetology, Department of Internal Medicine, Medical University of Graz, Auenbruggerplatz 15, 8036 Graz, Austria; 2 BioTechMed Graz, Graz, Austria; 3 CBmed GmbH- Center for Biomarker Research in Medicine, Graz, Austria; 4 Institute of Molecular Biosciences, NAWI Graz, University of Graz, Graz, Austria; 5 Field of Excellence BioHealth, University of Graz, Graz, Austria; 6 Core Facility Ultrastructure Analysis, Center for Medical Research (ZMF), Medical University of Graz, Graz, Austria; 7 Gottfried Schatz Research Center for Cell Signaling, Metabolism and Aging, Division of Cell Biology, Histology and Embryology, Medical University of Graz, Graz, Austria; 8 Joanneum Research Forschungsgesellschaft mbH HEALTH – Institute for Biomedicine and Health Sciences, Graz, Austria; 9 Division of Plastic, Aesthetic and Reconstructive Surgery, Medical University of Graz, Graz, Austria; 10 Joanneum Research Forschungsgesellschaft mbH COREMED – Cooperative Centre for Regenerative Medicine, Graz, Austria

**Keywords:** Type 1 diabetes, Autoimmunity

## Abstract

Spermidine is a natural polyamine which was shown to prolong lifespan of organisms and to improve cardiac and cognitive function. Spermidine was also reported to reduce inflammation and modulate T-cells. Autophagy is one of the mechanisms that spermidine exerts its effect. Autophagy is vital for β-cell homeostasis and autophagy deficiency was reported to lead to exacerbated diabetes in mice. The effect of spermidine in type 1 diabetes pathogenesis remains to be elucidated. Therefore, we examined the effect of spermidine treatment in non-obese diabetic (NOD) mice, a mouse model for type 1 diabetes. NOD mice were given untreated or spermidine-treated water ad libitum from 4 weeks of age until diabetes onset or 35 weeks of age. We found that treatment with 10 mM spermidine led to higher diabetes incidence in NOD mice despite unchanged pancreatic insulitis. Spermidine modulated tissue polyamine levels and elevated signs of autophagy in pancreas. Spermidine led to increased proportion of pro-inflammatory T-cells in pancreatic lymph nodes (pLN) in diabetic mice. Spermidine elevated the proportion of regulatory T-cells in early onset mice, whereas it reduced the proportion of regulatory T-cells in late onset mice. In summary spermidine treatment led to higher diabetes incidence and elevated proportion of T-cells in pLN.

## Introduction

Spermidine is a natural polyamine that affects cellular functions and cell homeostasis. In general, polyamines bind and stabilize DNA and RNA, show antioxidant activities, regulate enzymatic reactions, and are involved in the regulation of translation^1,2^. Spermidine treatment has been shown to prolong the life span of yeast, flies, worms, mammalian cells and mice^3,4^ and to lead to cardioprotection^3^ and improved cognitive function^5^ in aging mice. Spermidine is also an important factor in the regulation of the immune system on various levels. While spermidine treatment has been shown to reduce the production of proinflammatory cytokines^6–8^ and inflammation by modulation of macrophages in various inflammation models^9,10^, it also showed activating and immune-enhancing functions on CD8+ T-cells^11^, regulatory T-cells (Tregs)^12^ and B-cells^13^, which delay immunosenescence in mice. In type 1 diabetes, T-cells are the main contributors to disease pathogenesis and both CD4+ and CD8+ T-cells have been associated with beta cell destruction in non-obese diabetic (NOD) mice^14^, with e.g. CD4+ T-cells recruiting macrophages and natural killer cells to the pancreatic islets^15,16^. Tregs counteract the pro-inflammatory T-cells to prevent diabetes development^17^. Some of the positive effects of spermidine treatment have been attributed to spermidine-induced autophagy^18^. In a type 2 diabetes mouse model, spermidine-induced autophagy led to improved glucose and insulin tolerance and reduced body weight^19^. In type 1 diabetes mouse models, autophagy-deficient mice had exacerbated chemically-induced diabetes compared to wild-type mice^19^ and impaired autophagy was shown in pancreatic islets^20^. Although under physiological conditions autophagy has been shown to be important in maintaining beta cell function in beta-cell specific knock-out models^21,22^ it is unknown whether beta cell function can be protected or restored by spermidine treatment. We thus aimed to study the role of spermidine treatment in the pathogenesis of type 1 diabetes in NOD mice. Spermidine treatment was administered to NOD mice via drinking water. We investigated the effect of spermidine on diabetes incidence, pancreatic inflammation, insulin granule homeostasis, pancreatic autophagy levels, and immune cell pool.

## Materials and methods

All methods were carried out in accordance with relevant guidelines and regulations.

### Animals and diet

NOD/ShiLtJ strain (NOD mice) was purchased from Charles River and inbred under specific pathogen free conditions in the animal facility at the Medical University of Graz. All mice experiments were approved by the Federal Ministry Republic of Austria Education, Science and Research (TVA number: BMWFW-66.010/0142-WF/V/3b/2017). All mice experiments were compliant to ARRIVE guidelines. Mice were maintained at 12 h:12 h dark:light cycle in individual ventilated cages under pathogen free conditions. NOD mice had ad libitum access to standard diet and water. Water consumption was measured twice a week, body weight (BW) and food consumption were measured once a week. To assess diabetes onset non-fasting blood glucose obtained by tail puncture was measured by glucometer (Accu Check, Roche, Switzerland) twice a week after 10 weeks of age. In two separate studies we assessed (a) the effects of 10 mM spermidine treatment on tissue polyamine levels in male NOD mice and (b) the effects of daily oral 10 mM spermidine treatment in female NOD mice. Sample size was determined based on literature^3,23^. Researchers were aware of the group allocation during mice studies, but they were blinded regarding group allocation during sample analyses. Health status was regularly assessed during the studies by researchers and the staff veterinarian of Medical University of Graz.

**Figure 1:**
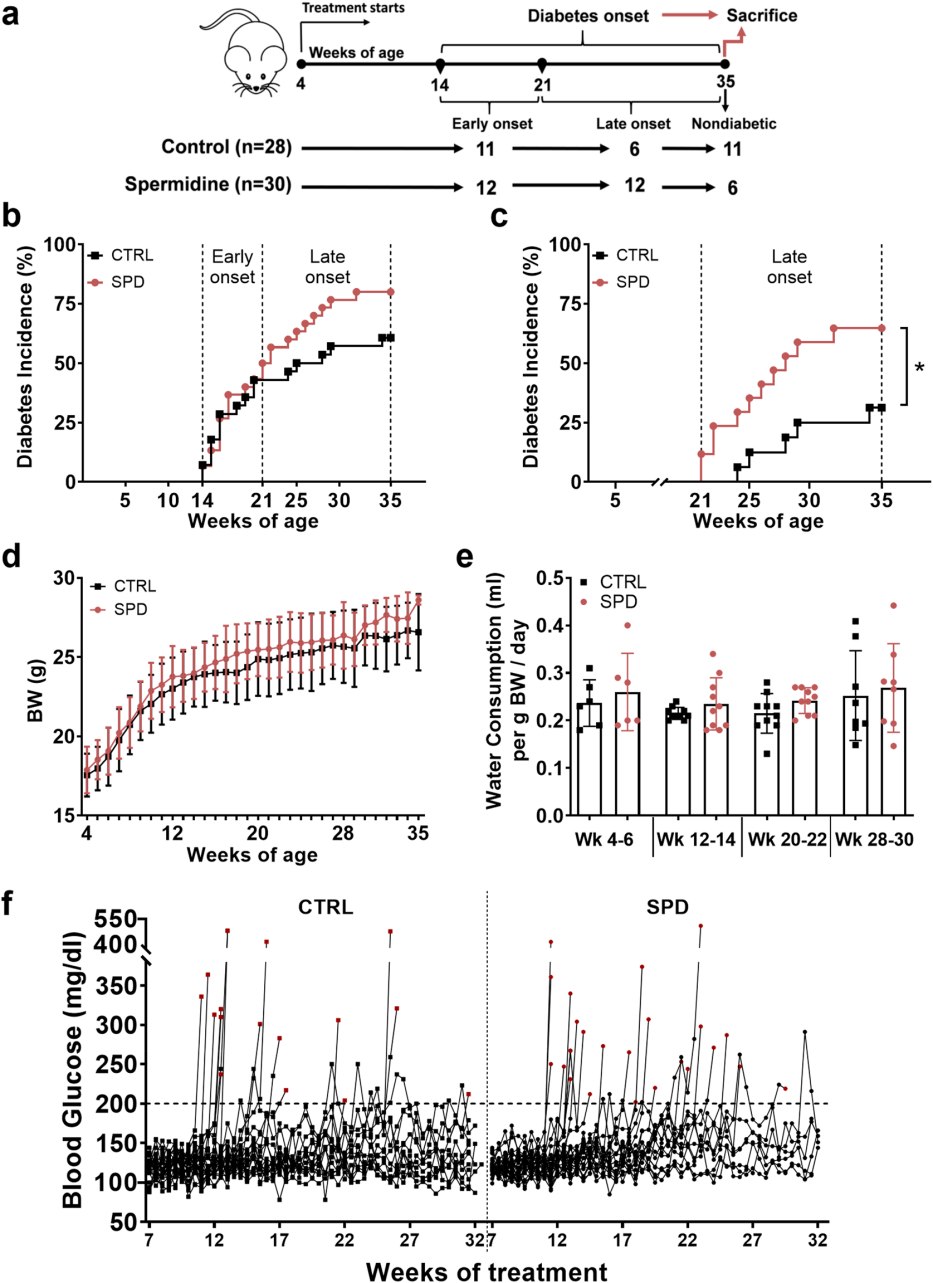
Daily oral spermidine treatment led to higher diabetes incidence in female NOD mice. (**a**) Experimental design. Diabetes incidence analyzed (**b**) throughout the study and (**c**) from week 21 to week 35. (**d**) BW gain (week 4–35), ctrl (n = 28) and 10 mM spd (n = 30). (**e**) Water consumption (week 4–35), each bar graph represents 4–6 independent measurements from two cages. (**f**) Individual blood glucose levels measured from 7 to 32 weeks of treatment, red squares or circles show blood glucose level at diabetes onset. Mice with blood glucose levels over 200 mg/dl in two consecutive measurements were determined as diabetic. (**b**–**e**) Black squares indicate ctrl mice and red circles indicate spd mice. Mantel-cox log rank test for analysis of diabetes incidence, two-way ANOVA with Sidak’s post-hoc test for analysis of BW and blood glucose throughout the study and unpaired Student’s *t*-test for analysis of water consumption between ctrl and spd groups were used as statistical analysis. p* < 0.05.

Each litter was equally divided and randomly assigned to either control or spermidine treatment at 4 weeks of age. Mice were given untreated water or 10 mM spermidine-treated water ad libitum. Spermidine was reconstituted in 1 M sterile water and stored in aliquots at − 80 °C for maximum of 3 months. Water bottles were freshly prepared containing 10 mM spermidine and changed twice a week between 2 and 5 pm. In the first study, 8 control (ctrl) male mice and 10 spermidine (spd) treated male mice were monitored for 28 days and sacrificed at day 29. In the second study, 30 ctrl and 30 spd female mice were treated from 4 weeks of age until diabetes onset or 35 weeks of age (Fig. 1a). 2 ctrl mice had to be excluded from all the analysis because one mouse was severely sick and one mouse was found dead. Mice were determined as diabetic when two consecutive blood glucose measurements were above 200 mg/dl in non-fasting state. Mice were sacrificed as nondiabetic at 35 weeks of age or as diabetic when diabetes onset was diagnosed. Diabetic mice were sacrificed on the same day as the second consecutive blood glucose measurement. Flow cytometric analysis was not performed when one blood glucose measurement was higher than 600 mg/dl. Mice were anesthetized by intraperitoneal injection of ketamine (AniMedica, Germany) and xylazine (Bayer, Germany) solution (0.1 ml/10 g BW). Anesthetized mice were sacrificed by cervical dislocation.

### Histology

Pancreata were fixed in 10% formalin (pH 6.90–7.10) and embedded in paraffin. Tissue sections (2 µm) were cut and selected 50 µm apart to avoid overlapping of islets for hematoxylin and eosin staining, which was accomplished except for 1 big islet. Five slides and a minimum of 14 islets per mouse were analyzed in aged-matched ctrl and spd diabetic mice with an age from 14 to 29 weeks. Images were captured with an Aperio ScanScope digital slide scanner (Leica Biosystems, Germany) at 40-fold magnification. Islets were counted and classified in a blinded fashion by two independent observers. Immune cell infiltration in islets was classified as published before^24^: grade 0: no infiltration, grade 1: peri-insulitis to 10% infiltration, grade 2: 10–50% infiltration, grade 3: 50–75% infiltration, grade 4: 75–100% infiltration.

### Electron microscopy

Pancreata were fixed in in 2.5% (wt/vol) glutaraldehyde and 2% (wt/vol) paraformaldehyde in 0.1 M cacodylate buffer, pH 7.4, for 2 h, post-fixed in 1% (wt/vol) osmium tetroxide for 2 h at room temperature. After dehydration in graded series of ethanol, tissues were infiltrated (ethanol and TAAB epoxy resin, pure TAAB epoxy resin) and placed in TAAB epoxy resin (8 h), transferred into embedding moulds, and polymerized (48 h, 60 °C). Ultrathin sections (70 nm) were cut with a UC 7 Ultramicrotome (Leica Microsystems, Vienna, Austria) and stained with lead citrate (5 min) and platin blue (15 min). Photomontages (Serial EM) were taken using a Tecnai G2 20 transmission electron microscope (FEI, Eindhoven, Netherlands) with a Gatan Ultrascan 1000 charge coupled device (CCD) camera (− 20 °C; acquisition software Digital Micrograph; Gatan, Munich, Germany). Acceleration voltage was 120 kV. Image calculations (granules and cluster counting) were done with Image J Software (ImageJ 1.52p Java 1.8.0.172)^25^.

### ELISA

Pancreas samples were freshly collected in extraction buffer (98.5 mL 75% EtOH, 1.5 mL 38% HCl). Samples were homogenized by ULTRA-TURRAX Homogenizer T10 standard (IKA-Werke, Germany) for 30 s (3 × 10 s, stand 3–4) on ice and then incubated overnight at 4 °C. Then each sample was sonicated by Ultrasonic Processor (UP50H, Hielscher Ultrasonics, Germany) for 30 s and centrifuged for 20 min at 400 × *g*. Homogenates were collected in a new Eppendorf tube and stored at − 20 °C. For ELISA experiments, samples were measured in duplicates. Mouse ultrasensitive insulin ELISA plates were purchased from Alpco (USA). The measurements were performed according to manufacturer’s guidelines. Samples were measured with Spectrostar Omega (BMG Lab Tech, Germany).

### Immunoblot analysis

Tissues were pulverized by mortar and pestle on dry ice and then lysed (45 min, on ice) with lysis buffer (150 mM NaCl, 1 mM EGTA, 1 mM EDTA, 1% Triton X-100 and 20 mM Tris pH. 7.5) in the presence of Halt™ Protease and Phosphatase Inhibitor Cocktail (Thermo Fisher Scientific, USA). Total protein concentration was determined by BCA Protein Assay Kit (Thermo Fisher Scientific, USA). Equal amounts of tissue lysates were heated at 95 °C (5 min) and loaded on 4–20% TGX precast gels (Biorad, USA) and transferred to polyvinylidene difluoride membranes (Biorad, USA) by Trans-Blot Turbo transfer system (Biorad, USA). The blots were probed for LC3 (1:1000, # 2775), p62 (1:1000, # 5114) and Beclin1 (1:1000, # 3738) antibodies (all from CST, USA) and goat anti-rabbit HRP-linked secondary antibody (1:5000, # 7074, CST, USA) and developed with ECL (Biorad, USA). Blots were stained with Coomassie brilliant blue (Biorad, USA) for normalization. The bands were detected by Biorad Chemidoc Touch imaging system (Biorad, USA) and analyzed by Image Lab software (version 6.0.1., RRID:SCR_014210, Biorad, USA). Each gel was run with positive control and pooled sample as an internal control. Nondiabetic mice served as comparison group, while diabetic mice were the test groups.

### Liquid chromatography- tandem mass spectrometry

For polyamine extraction, snap-frozen tissues were pulverized on dry ice using mortar and pestle and subjected to 5% trichloroacetic acid (TCA) extraction using 20–25 mg pulverized tissue or 20 µl whole blood and a final extract volume of 750 µl. Quantitative HPLC–MS/MS-based determination of spermidine, putrescine, ornithine and spermine was performed as described before by employing stable-isotope labeled internal standards^26^.

Samples were incubated on ice (1 h) and centrifuged at 10,000 g at 4 °C (10 min). 150 µl supernatant was mixed with 800 µl double distilled water, 125 µl sodium carbonate buffer (1 M, pH 9) and 25 µl isobutyl chloroformate, then incubated at 35 °C (15 min). The solution was centrifuged at 10,000 g (1 min) and the resulting supernatant was measured in the Ultimate 3000 HPLC system (Thermo Fisher Scientific, USA) coupled to a triple-quadrupole mass spectrometer, a Quantum TSQ Ultra AM controlled by Xcalibur Software version 4.0 (both Thermo Fisher Scientific, USA).

### Flow cytometry

Spleen and pancreatic lymph nodes (pLN) were collected freshly in cooled RPMI media (pH 7.4, Thermo Fisher Scientific, USA), supplemented with 5% FBS (Thermo Fisher Scientific, USA) and 1% penicillin/ streptomycin (Thermo Fisher Scientific, USA) and stored on ice. Blood was collected by cardiac puncture using 150 U/ml lithium heparin (Sigma-Aldrich, USA) flushed syringes in lithium-heparin coated tubes (Sarstedt, Germany). Blood and tissues were processed immediately to obtain single cell suspensions. Whole blood was processed for FACS analysis including a red blood cell lysis step with ACK lysis buffer (Lonza, Switzerland). Spleen and pLN were minced and crushed through 100 µm cell strainers (BD Biosciences, USA), and centrifuged at 400 g (5 min) at room temperature and resuspended in PBS for antibody staining. Spleen cells were additionally treated with ACK lysis buffer (Lonza, Switzerland) to remove red blood cells. The isolated cell suspensions from pLN, blood and spleen were incubated with Fc blocking reagent (Thermo Fisher Scientific, USA) and stained with monoclonal fluorescence-conjugated antibodies and fixable viability dye (Thermo Fischer, USA). Antibodies were purchased from Miltenyi Biotech (Germany); CD4 (GK1.5), CD25 (REA568), CD44 (IM7.8.1), CD62L (REA828), FoxP3 (REA788), from Thermo Fisher Scientific (USA); CD3 (145-2C11), CD8a (53–6.7) and BD Biosciences (USA); CD45 (30-F11), CD25 (PC61), CTLA4 (UC10-4F10-11). For additional intracellular staining, cells were fixed and permeabilized with transcription factor buffer set (BD Biosciences, USA) and stained with FoxP3 (REA788) and CTLA4 (UC10-4F10-11) antibodies. Appropriate isotype controls and fluorescence minus one control (FMO) tubes were included for identification of positive signals. Cells were analyzed using a BD LSR Fortessa SORP device and Diva software (version 8.0.1; BD Biosciences, USA).

All immune cell populations were always gated for singularity (FSC-H vs FSC-A) and viability. Then T cell populations were gated accordingly: CD4+ T-cells (CD45+, CD3+, CD4+), CD8+ T-cells (CD45+, CD3+, CD8+), naïve CD4+ T-cells (CD45+, CD3+, CD4+, CD44(−), CD62L+), effector memory CD4+ T-cells (CD45+, CD3+, CD4+, CD44+, CD62L(−)), central memory CD4+ T-cells (CD45+, CD3+, CD4+, CD44+, CD62L+), naïve CD8+ T-cells (CD45+, CD3+, CD8+, CD44(−), CD62L+), effector memory CD8+ T-cells (CD45+, CD3+, CD8+, CD44+, CD62L(−)), central memory CD8+ T-cells (CD45+, CD3+, CD8+, CD44+, CD62L+), FoxP3 T-cells (CD45+, CD3+, CD4+, FoxP3+), total regulatory T-cells (CD45+, CD3+, CD4+, FoxP3+, CD25+), activated regulatory T-cells (CD45+, CD3+, CD4+, FoxP3+, CD25+, CTLA4+).

### Statistical analysis

Initial analyses were done by comparing ctrl and spd groups. For more detailed analyses, statistics were based on grouping the mice as early onset (< 21 weeks of age) and late onset (≥ 21 weeks of age) (Fig. 1b). Data is presented as mean ± SD and was tested for normality using Shapiro–Wilk test. Diabetes incidence was evaluated by Mantel-Cox log rank test. Statistical significance between two groups was determined using Mann–Whitney U test or unpaired two-tailed unpaired Student’s *t*-test. Two-way ANOVA was used for multiple comparisons. Graphs and statistics were prepared in GraphPad Prism version 8.0.2 (GraphPad Software, San Diego, California USA, www.graphpad.com). Results were considered significant for p* < 0.05, p** < 0.01, p*** < 0.001, p**** < 0.0001.

## Results

### Spermidine treatment leads to higher diabetes incidence

We investigated the influence of daily oral spermidine treatment on type 1 diabetes in female NOD mice. 17 mice from the ctrl group and 24 mice from the spd group were diagnosed as diabetic, and 11 mice from the ctrl group and 6 mice from the spd group remained nondiabetic at 35 weeks of age (Fig. 1a). Analysis of diabetes incidence until 35 weeks of age showed that ctrl and spd mice did not have a significant difference in diabetes incidence, although diabetes development in the ctrl and the spd group was similar until the mice were 21 weeks of age (Fig. 1b), at 21 weeks of age difference in diabetes incidence became apparent and significant. The spd group had a significantly higher diabetes incidence between 21 and 35 weeks of age (Fig. 1c).

BW at the beginning of the study was similar between the ctrl and spd group (Fig. 1d and Supplementary Fig. 1a). BW gain as well as water and food consumption were similar between ctrl and spd groups throughout the study (Fig. 1d, e and Supplementary Fig. 1b). Blood glucose levels between ctrl and spd group remained similar throughout the study (Fig. 1f, Supplementary Fig. 1c).

### Spermidine does not alter insulitis in pancreatic islets

**Figure 2:**
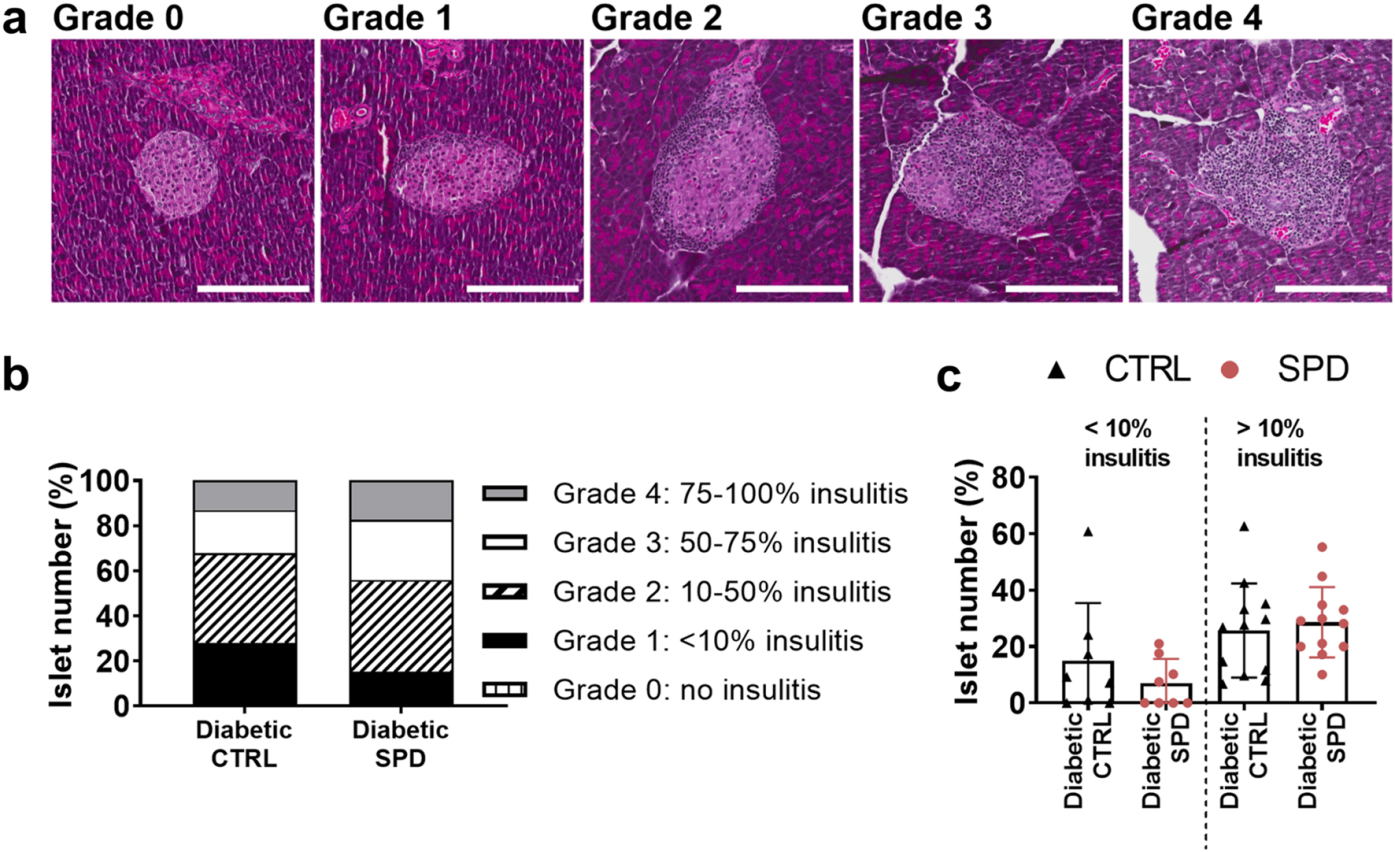
Daily oral spermidine treatment did not alter insulitis in pancreatic islets of female diabetic mice. (**a**) Insulitis grading map: grade 0 (no insulitis), grade 1 (peri insulitis to 10% insulitis), grade 2 (10- 50% insulitis), grade 3 (50- 75% insulitis), grade 4 (75–100% insulitis), scale bar: 200 µm, 20-fold magnification. (**b**) Number of islets per grade normalized to total number of islets in each mouse. (**c**) Percentage of islets having < 10% insulitis and > 10% insulitis analyzed in diabetic ctrl mice and diabetic spd mice in accordance to insulitis grading (2 datapoints per mouse in each group), black triangles show diabetic ctrl mice and red circles show diabetic spd mice. Diabetic ctrl mice (n = 4) and diabetic spd mice (n = 4). Two-way ANOVA with Sidak’s post-hoc test to compare respective grades between ctrl and spd groups and Mann–Whitney U test to compare ctrl and spd groups were used as statistical analysis.

To study the effect of spermidine treatment on pancreatic inflammation, we examined insulitis in pancreatic islets of age-matched ctrl and spd diabetic mice aged 14–29 weeks (Fig. 2a). The insulitis levels were not statistically different in the pancreatic islets of diabetic ctrl and spd mice (Fig. 2b, c). Diabetic spd mice had slightly less grade 1 insulitis and slightly more grade 3 and grade 4 insulitis compared to diabetic ctrl mice (Fig. 2b). Similarly, in an alternative graph depiction, diabetic spd mice had slightly less lightly inflamed islets (< 10%) while diabetic ctrl and diabetic spd mice had similar percentage of heavily inflamed islets (> 10%) (Fig. 2c).

### Spermidine does not alter insulin granule homeostasis in nondiabetic mice

**Figure 3:**
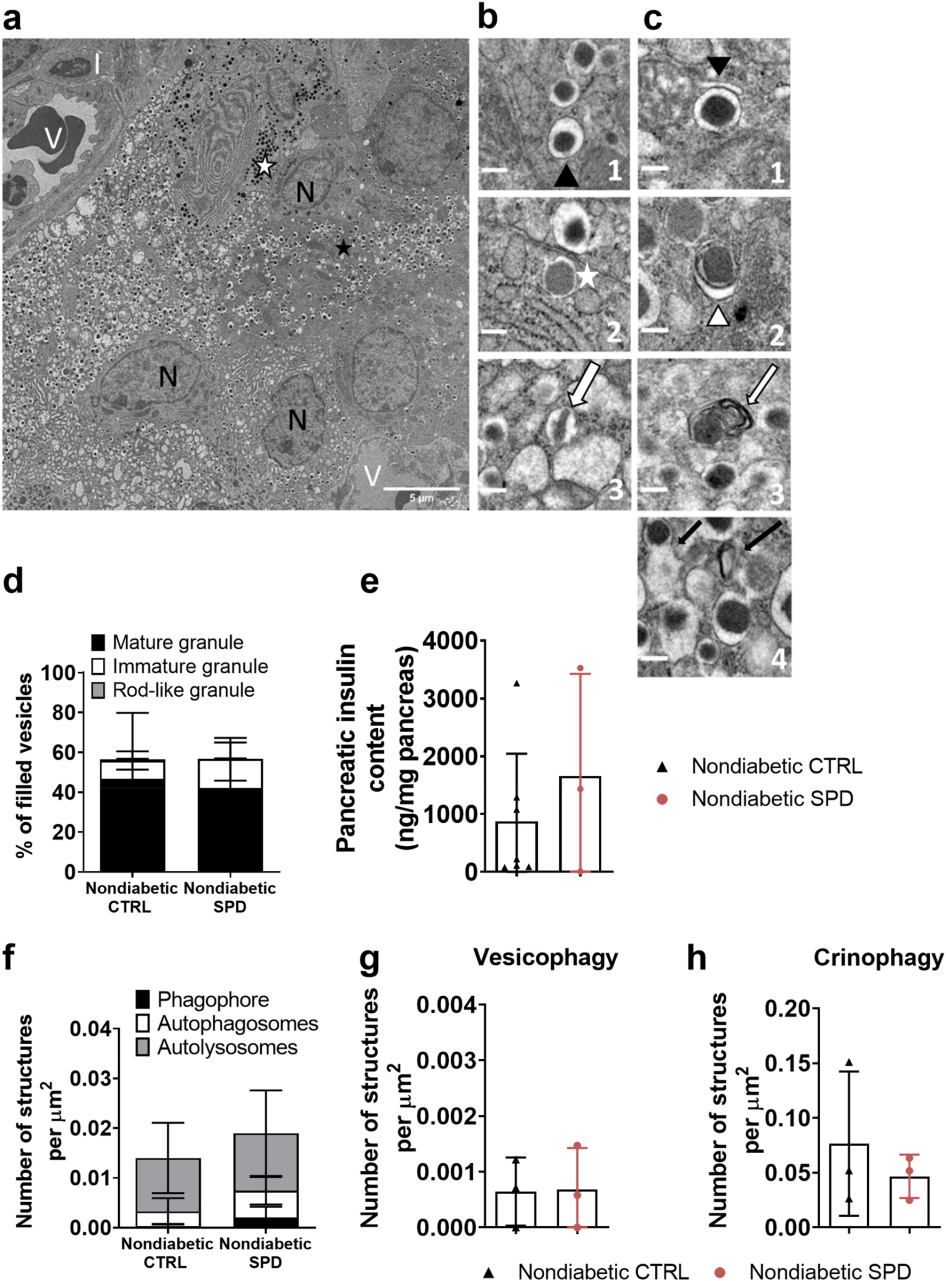
Daily oral spermidine treatment did not alter insulin granule homeostasis in female nondiabetic mice. (**a**) A stitched micrograph including 25 single micrographs (V: vessel, N: nucleus, I: immune cell), scale bar: 5 µm. (**b1**) Black arrowhead indicates mature granules with a condensed core containing insulin. (**b2**) White asterisk marks immature transforming granules with a white halo. (b3) White arrow indicates rod-like insulin granules—crystallized structure surrounded by a bright halo, scale bar: 0.5 µm. (**c1**) Black arrowhead indicates a phagophore (dilated membrane) enwrapping a mature granule with a dark core. (**c2**) White arrowhead marks autophagosome (double bilayer) surrounding a mature granule. (**c3**) Autolysosome: white arrow indicates enclosed digested granule without a halo—membrane stacks within the autolysosome. (**c4**) Crinophagy: black arrows indicate a fusion of mature granules with electron-bright lysosomes, scale bar: 0.5 µm. (**d**) Insulin granule types normalized to filled vesicles. (**e**) pancreatic insulin content measured by ELISA (ctrl n = 7, spd n = 3). (**f**) Number of autophagy pathway structures (phagophore, autophagosome, autolysosome) per µ${\mathrm{m}}^{2}$. (**g**) Number of vesicophagic vesicles per µm^2^. (**h**) Number of crinophagic vesicles per µ${\mathrm{m}}^{2}$. Mice with blood glucose levels less than 200 mg/dl in two consecutive measurements were determined as nondiabetic at 35 weeks of age. Each datapoint represents the average of 3 islets from one mouse; area of 332 µ${\mathrm{m}}^{2}$–2272 µ${\mathrm{m}}^{2}$ from each islet was analyzed. Nondiabetic ctrl mice (n = 3) and nondiabetic spd mice (n = 3). Data is shown as mean ± SD. Mann–Whitney U test was used as statistical analysis.

To study the effect of long-term treatment with spermidine, we examined the total pancreatic insulin content by ELISA and insulin granules in beta cells of pancreatic islets by electron microscopy in nondiabetic ctrl and nondiabetic spd mice at week 35 (Fig. 3a–c). Daily oral spermidine treatment did not alter the percentage of mature, immature and rod-like granules between the nondiabetic ctrl mice and the nondiabetic spd mice (Fig. 3d). Similar to the unchanged percentage of insulin granules, spermidine treatment did not alter pancreatic insulin content measured by ELISA (Fig. 3e). Spermidine treatment did not change the number of autophagy pathway structures which were identified as phagophore, autophagosome and autolysosome (Fig. 3f). The number of double membrane structures including insulin granules (vesicophagy) and number of crinophagic vesicles were similar in nondiabetic ctrl mice and nondiabetic spd mice (Fig. 3g, h). We also described and counted some granule structures consisting of fusion of immature and mature granules or fusion of mature granules. There was no significant difference in the percentage of fusion structures between the nondiabetic ctrl mice and the nondiabetic spd mice (Supplementary Fig. 2a, b).

### Spermidine increases autophagy in the pancreas of diabetic mice

**Figure 4:**
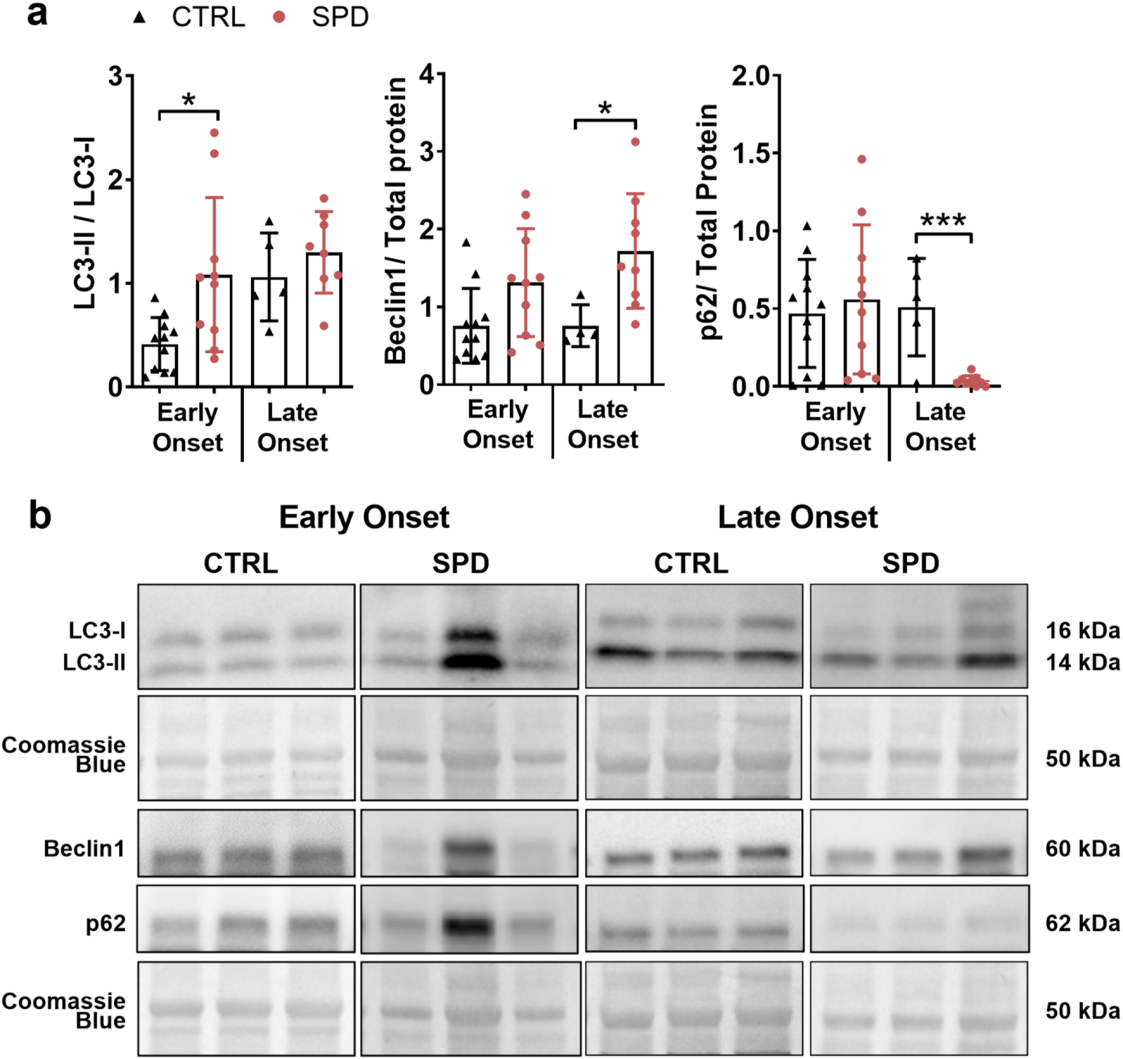
Daily oral spermidine treatment increased autophagy in female late onset mice. Diabetic mice were grouped as early onset (< 21 weeks of age) and late onset (≥ 21 weeks of age). Samples were probed for LC3, Beclin1, p62 and stained with Coomassie brilliant blue for total protein in immunoblot. (**a**) LC3-II to LC3-I ratio, Beclin1 normalized to total protein, p62 normalized to total protein. (**b**) Representative cropped images of immunoblots. Early onset ctrl mice (n = 11), early onset spd mice (n = 10), late onset ctrl mice (n = 4–5), late onset spd mice (n = 8–9). Black triangles indicate ctrl mice and red circles indicate spd mice. Data is shown as mean ± SD. Mann–Whitney U test or unpaired Student’s *t*-test was used as statistical analysis to compare ctrl and spd groups. p* < 0.05, p*** < 0.001.

We investigated whether autophagy markers were altered by spermidine treatment in the pancreas by monitoring protein expression of LC3-I, LC3-II, p62 and Beclin1 in immunoblot (Fig. 4). Representative immunoblots are shown as cropped images in Fig. 4b and full images of immunoblots are shown in Supplementary Fig. 10.

Diabetic spd mice had increased LC3-II to LC3-I ratio (1.8-fold, *p* < 0.01) and increased Beclin1 levels (2-fold, *p* < 0.001) compared to diabetic ctrl mice (Supplementary Fig. 3). Early onset spd mice had higher LC3-II to LC3-I ratios relative to early onset ctrl mice (2.6-fold, *p* < 0.01) (Fig. 4a). Late onset spd mice had elevated Beclin1 levels (2.4-fold, *p* < 0.05) and reduced p62 levels (15.3-fold, *p* < 0.05) compared to late onset ctrl mice (Fig. 4a).

### Spermidine treatment alters tissue polyamine levels

**Figure 5:**
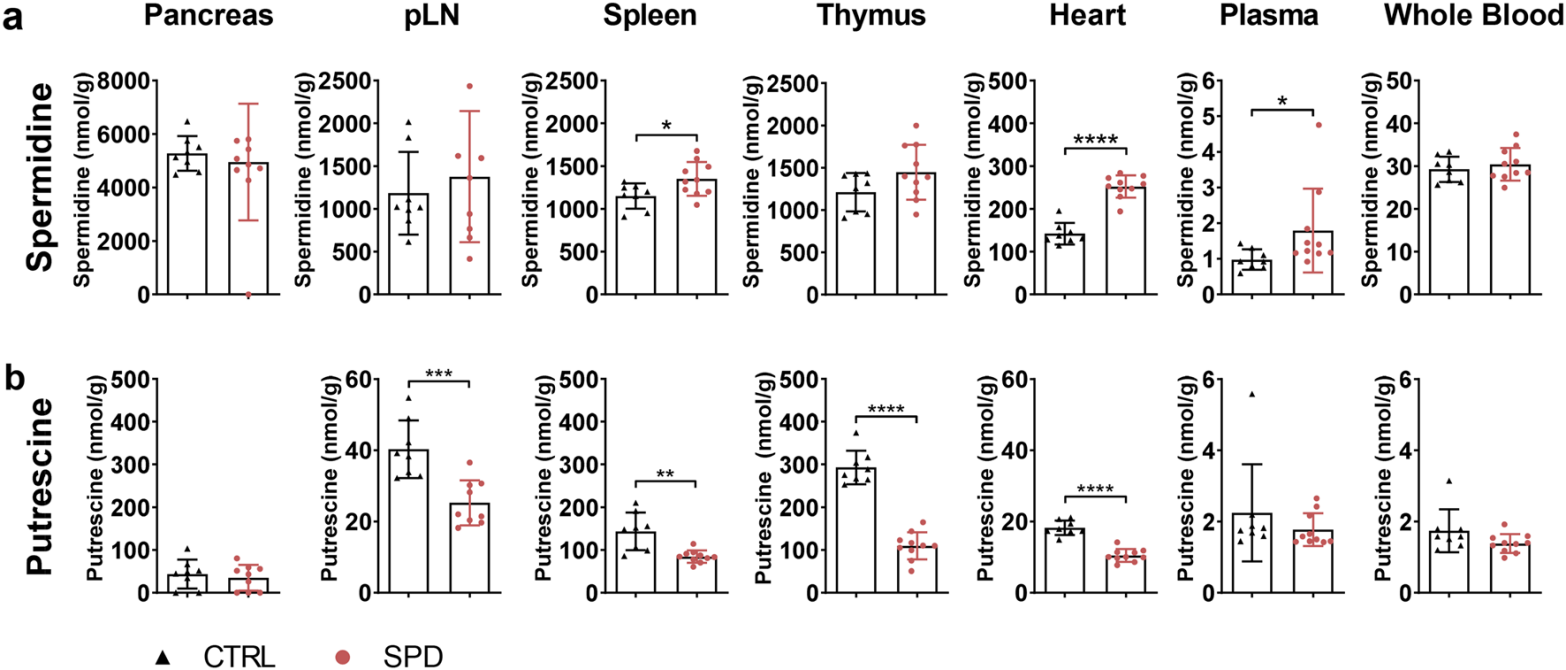
Daily oral spermidine treatment enhanced endogenous spermidine levels in male NOD mice. (**a**) Spermidine and (**b**) putrescine levels were analyzed in pancreas, pLN, spleen, thymus, heart, plasma and whole blood after 4 weeks of 10 mM spermidine treatment. Black triangles show ctrl mice (n = 8) and red circles show spd mice (n = 10). Data is shown as nmol/g and mean ± SD. Mann–Whitney U test or unpaired Student’s *t*-test was used as statistical analysis. p* < 0.05, p** < 0.01, p*** < 0.001, p**** < 0.0001.

To examine the effect of 10 mM spermidine treatment on tissue polyamine levels, we measured the endogenous levels of spermidine, its precursors putrescine, ornithine and its downstream metabolite spermine in ctrl and spd NOD nondiabetic male mice. Spermidine treatment significantly increased the endogenous spermidine levels in spleen (1.2-fold, *p* < 0.05), heart (1.8-fold, *p* < 0.0001) and plasma (1.8-fold, *p* < 0.05) (Fig. 5a). There was no significant accumulation of spermidine in pancreas, pLN, thymus and whole blood (Fig. 5a). Spermidine treatment significantly reduced putrescine levels in pLN (1.6-fold, *p* < 0.001), spleen (1.7-fold, *p* < 0.01), thymus (2.7-fold, *p* < 0.0001) and heart (1.7-fold, *p* < 0.0001) (Fig. 5b), but did not alter ornithine and spermine levels (Supplementary Fig. 4).

### Spermidine alters immune cell populations in diabetic mice

**Figure 6:**
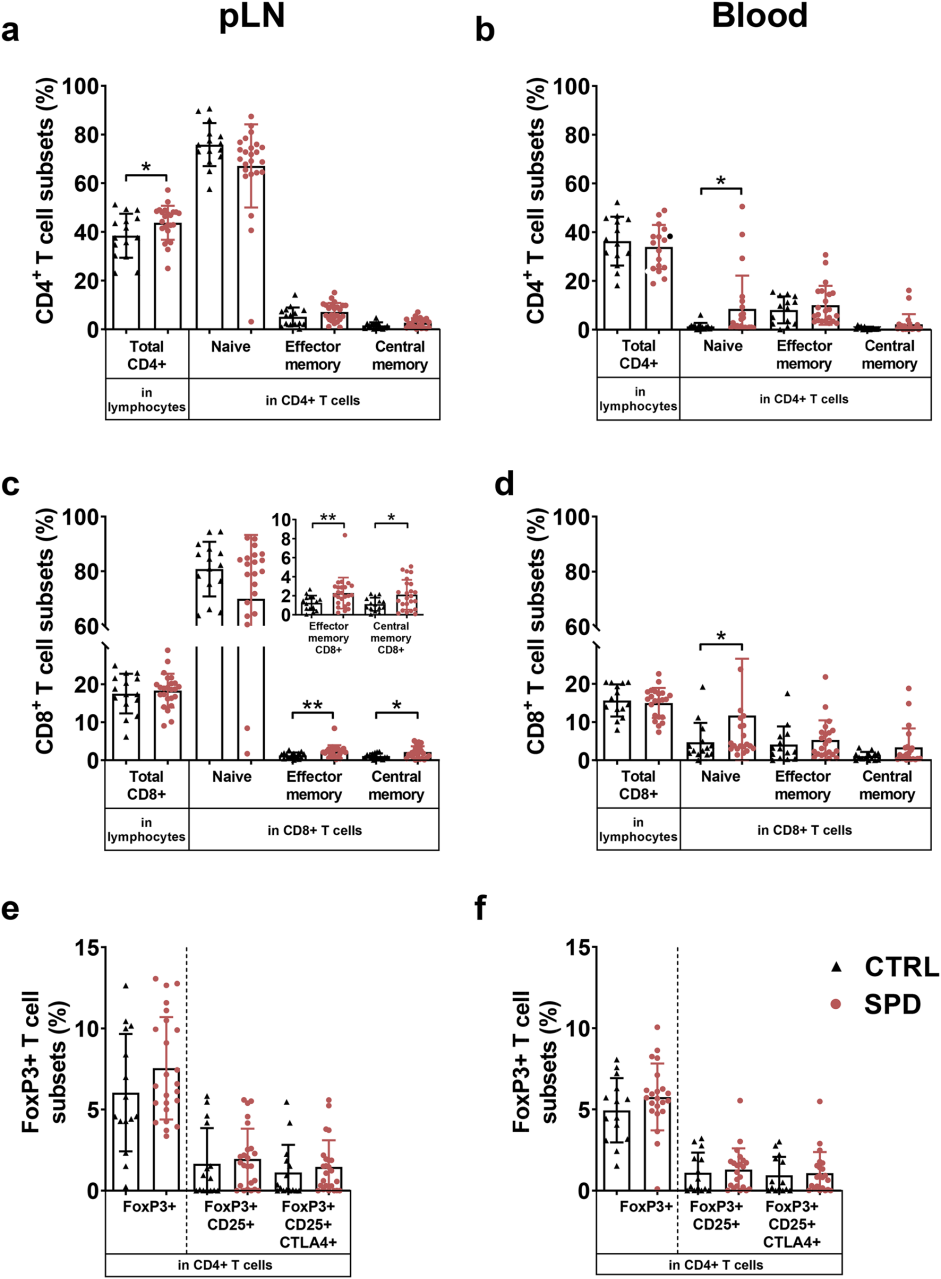
Daily oral spermidine treatment increased the proportion of CD8+ T-cell subsets in female diabetic mice. Total CD4+ T-cells, naïve CD4+ T-cells, effector memory CD4+ T-cells and central memory CD4+ T-cells in (**a**) pLN and (**b**) blood, total CD8+ T-cells, naïve CD8+ T-cells, effector memory CD8+ T-cells and central memory CD8+ T-cells in (**c**) pLN and (**d**) blood and FoxP3+ T-cells, FoxP3+ CD25+ Tregs, FoxP3+ CD25+ CTLA4+ Tregs in (**e**) pLN and (**f**) blood were examined. Black triangles show diabetic ctrl mice; for pLN (n = 15), for blood (n = 14) and red circles show diabetic spd mice; for pLN (n = 24), for blood (n = 22). Data is shown as mean ± SD. Mann–Whitney U test or unpaired Student’s *t*-test was used as statistical analysis to compare ctrl and spd mice. p* < 0.05, p** < 0.01.

Mice diagnosed as diabetic between age of 14 and 35 weeks were analyzed for their immune cell profile in pLN, blood and spleen. Gating strategy is shown in Supplementary Fig. 5. In pLN, diabetic spd mice had an increased proportion of total CD4+ T-cells (1.1-fold, *p* < 0.05), effector memory CD8+ T-cells (1.8-fold, *p* < 0.01) and central memory CD8+ T-cells (1.9-fold, *p* < 0.05) compared to controls (Fig. 6a, c). In blood, diabetic spd mice had a higher proportion of naïve CD4+ T-cells (6.5-fold, *p* < 0.05) and naïve CD8+ T-cells (2.5-fold, *p* < 0.05) compared to diabetic ctrl mice (Fig. 6b, d). In pLN and blood, diabetic spd and ctrl mice had similar proportion of FoxP3+ T-cells, FoxP3+ CD25+ Tregs and FoxP3+ CD25+ CTLA4+ Tregs (Fig. 6e, f). In spleen, diabetic ctrl and diabetic spd mice had similar proportion of CD4+ T-cell subsets and CD8+ T-cell subsets (Supplementary Fig. 6).

To investigate the differential effect of spermidine on diabetes incidence, we analyzed the diabetic mice based on their age at diabetes onset. Diabetic mice were grouped as early onset (< 21 weeks of age) and late onset (≥ 21 weeks of age) based on the statistical difference in the diabetes incidence. In pLN, early onset spd mice had increased proportion of total CD4+ T-cells (1.2-fold, *p* < 0.05) compared to early onset ctrl mice (Supplementary Fig. 7a). In blood, early onset spd mice had decreased proportion of total CD4+ T-cells (1.2-fold, *p* < 0.05) and CD8+ T-cells (1.3-fold, *p* < 0.0.5) compared to early onset ctrl mice (Supplementary Figs. 7b and 8b). In blood, late onset spd mice had increased proportion of total CD8+ T-cells (1.5-fold, *p* < 0.0.5) compared to late onset ctrl mice (Supplementary Fig. 8b). In spleen, early and late onset mice had similar proportion of CD4+ T-cell subsets and CD8+ T-cell subsets (Supplementary Figs. 7c and 8c).

**Figure 7:**
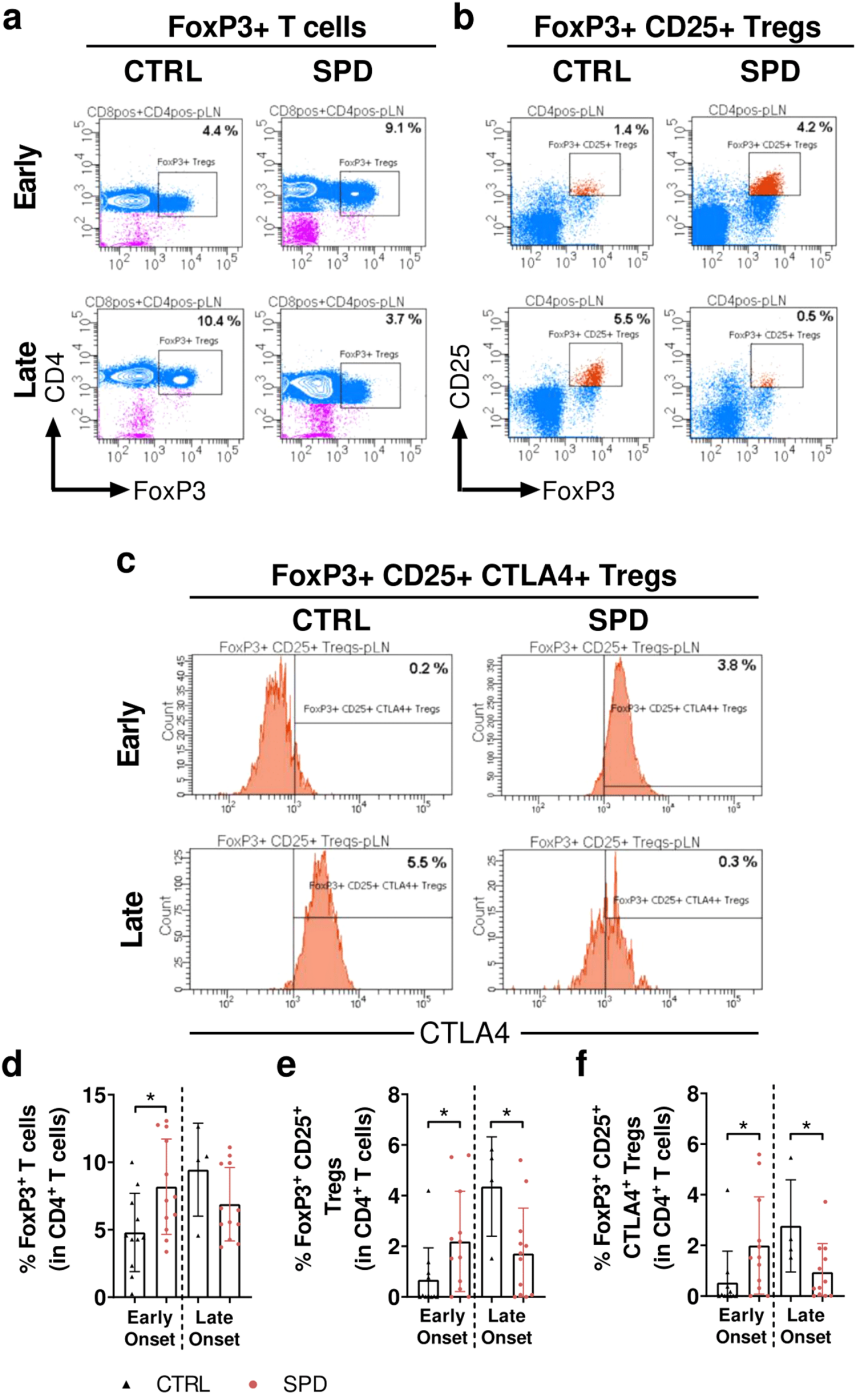
Daily oral spermidine treatment increased the proportion of Tregs in female early onset spd mice and decreases the proportion of Tregs in late onset spd mice. Representative images of gating strategy are shown in (**a**) FoxP3+ T-cells, (**b**) FoxP3+ CD25+ Tregs and (**c**) FoxP3+ CD25+ CTLA4+ Tregs (**d**) Proportion of FoxP3+ T-cells, (**e**) FoxP3+ CD25+ Tregs and (**f**) FoxP3+ CD25+ CTLA4+ Tregs were examined in pLN of early and late onset mice. Diabetic mice were grouped as early onset (< 21 weeks of age) and late onset (≥ 21 weeks of age). Early onset ctrl mice (n = 11), early onset spd mice (n = 12), late onset ctrl mice (n = 4), late onset spd mice (n = 12). Black triangles indicate diabetic ctrl mice and red circles indicate diabetic spd mice. Data is shown as mean ± SD. Mann–Whitney U test or unpaired Student*’*s *t*-test was used as statistical analysis to compare ctrl and spd mice. p* < 0.05.

While spermidine treatment increased the proportion of FoxP3+ T-cells and Tregs in pLN of early onset spd mice, this proportion was decreased in pLN of late onset spd mice (Fig. 7). In pLN, early onset spd mice had increased proportion of FoxP3+ T-cells (1.7-fold, *p* < 0.05), FoxP3+ CD25+ Tregs (3.2-fold, *p* < 0.05) and FoxP3+ CD25+ CTLA4+ Tregs (3.8-fold, *p* < 0.05), while late onset spd mice had reduced proportions of FoxP3+ CD25+ Tregs (2.6-fold, *p* < 0.05) and FoxP3+ CD25+ CTLA4+ Tregs (3-fold, *p* < 0.05) (Fig. 7). In blood and spleen, the proportion of FoxP3+ T-cells and Tregs was similar between ctrl and spd groups in early and late onset mice (Supplementary Fig. 9).

## Discussion

We assessed the effect of spermidine treatment in NOD mice, where ~ 50% of individuals spontaneously develop a type 1 diabetes phenotype at a young age. Daily oral spermidine treatment in NOD mice led to a higher diabetes incidence, organ-specific accumulation of spermidine, increased T-cells but no change in insulitis levels. Although spermidine treatment was shown to modulate immune cells in various inflammation models^10–13^, no previous study has directly investigated the role of spermidine treatment in type 1 diabetes pathogenesis.

We found that daily oral spermidine treatment led to a significantly higher diabetes incidence in spermidine-treated mice compared to control mice. In line with our results, a previous study showed that polyamine depletion delayed diabetes onset in NOD mice via diminishing eIF5a hypusination^27^. Hypusinated eIF5a was also shown to be elevated in pancreas samples of patients with type 1 diabetes and pancreas samples of NOD mice compared to controls^28^. Similarly, inhibition of eIF5a delayed diabetes and led to an increased number of Tregs in pancreas, pLN, and spleen in a humanized type 1 diabetes mouse model^29^. Overall, inhibition of eIF5a hypusination is suggested to be protective in mouse models of type 1 diabetes. Since spermidine has been shown to increase eIF5a hypusination^13^, one possible reason for spermidine-induced higher diabetes incidence in our study could be spermidine-induced increased eIF5a hypusination.

Although we observed higher diabetes incidence with spermidine treatment, spermidine did not worsen insulitis in diabetic mice and did not alter pancreatic insulin content and the insulin granule pool in nondiabetic mice. Previous studies have shown that a depletion of polyamines led to reduced insulin content by inhibition of spermidine synthase. Spermidine synthase inhibition reduced endogenous spermidine levels leading to reduced Ca^+2^ levels and subsequently diminished insulin content^30^. An addition of exogenous spermidine reversed the Ca^+2^ levels and insulin content^30^. Similarly reduced spermine and spermidine levels due to overexpression of N-acetyltransferase in-vivo led to reduced insulin secretion evidenced by impaired glucose-induced insulin tolerance^31^. Although previous studies suggested that increased spermidine levels might improve insulin homeostasis, our data did not show an altered insulin content in NOD mice due to spermidine treatment.

Since one recent study suggested that constant and long-term autophagy induction in mice challenged with high-fat diet leads to a reduced insulin granule pool in an autophagy-dependent manner^32^, the effect of spermidine treatment on insulin granules was investigated in nondiabetic NOD mice. We observed that spermidine did neither alter the insulin granule pool nor crinophagy^33^ indicating no effect of spermidine. However, it remains possible that spermidine negatively affected the insulin granule pool in NOD mice before diabetes manifestation. The limitation of a poor comparability with studies using diabetic mice^32^ is going to be overcome by using pre-diabetic NOD mice in future studies and also by including autophagy-flux analysis that were not part of the current study.

Contrary to our findings in nondiabetic NOD mice, we observed signs of increased autophagy levels in whole pancreas of diabetic mice after spermidine treatment. This data is in line with recent studies that showed spermidine-induced autophagy in-vitro and in-vivo in various animal models^18^. For further autophagy analyses we split diabetic NOD mice into an early onset and a late onset group based on statistically different diabetes incidences. Late onset spermidine-treated NOD mice had significantly higher autophagy levels in pancreas which did not protect them from diabetes probably due to autophagy-induced beta cell apoptosis which has also been described in a previous study which showed that increased autophagy leads to increased apoptosis^34^. In-vitro and in-vivo studies have shown that rapamycin-induced autophagy triggered beta cell apoptosis and caused glucose intolerance^34,35^. Rapamycin also diminished the positive effects of potential type 1 diabetes therapies by inducing glucose intolerance when administered in combination with these therapies in mice^36,37^. We interpret our autophagy estimates only cautiously as we did not assess autophagy flux using lysosome inhibitors (e.g. leupeptin).

To further investigate the higher diabetes incidence induced by spermidine treatment, we measured endogenous polyamines in various tissues. We found increased spermidine levels in spleen, heart, and plasma but not in pancreas in spermidine-treated mice. In both spermidine-treated and control mice, we observed the highest spermidine levels in pancreas similar to an earlier study^38^. Thus, we speculate that oral spermidine treatment significantly increased spleen, heart, and plasma levels of spermidine but not pancreas levels since already high spermidine levels in pancreas cannot be further increased by daily spermidine treatment. In line with the interpretation of halted spermidine biosynthesis^39^, we also found reduced putrescine levels in pLN, spleen, thymus and heart. Modulated polyamine levels in pLN and spleen encouraged us to further investigate the immune cell pool in these tissues.

Our data showed that spermidine treatment led to an increased proportion of effector memory and central memory CD8+ T-cells in pLN of diabetic NOD mice. In line with our results, a previous study showed that polyamines affect the number of T-cells in different animal models^11^. For instance, spermidine-treated aged mice had a higher number of memory CD8+ T-cells compared to control aged mice in response to an immunization protocol^11^. As reviewed earlier^40^, many studies have found that polyamine synthesis and T-cell proliferation are directly associated. Especially, activity and production of polyamine-synthesizing enzymes were reported to be elevated upon T-cell activation^40^.

Based on the diabetes incidence, sub-analysis of early and late onset mice showed that in early onset mice spermidine substantially increased the percentage of FoxP3+ T-cells, total Tregs and activated CTLA4+ Tregs in pLN while in late onset mice spermidine substantially reduced the percentage of total Tregs and activated CTLA4+ Tregs in pLN. An increase in Tregs is recognized as a marker for diabetes prevention and Tregs were reported to maintain peripheral tolerance by suppressing pro-inflammatory T-cells^17^. However, in our study an increased proportion of Tregs in early onset NOD mice did not change diabetes incidence, despite a reduction in CD4+ T-cells and CD8+ T-cells in blood. A recent in-vitro study showed that spermidine treatment elevated the proportion of FoxP3+ T-cells but it did not change the suppressive function of FoxP3+ T-cells^12^. Based on our data we suggest that an unchanged suppressive function despite spermidine-induced elevated number of Tregs is a possible explanation for unaltered diabetes incidence in early onset mice. Alternatively, the spermidine-induced increase in Tregs might not have been large enough to be able to suppress the increased inflammation due to spermidine-induced effector memory CD8+ T-cells and central memory CD8+ T-cells. Future studies will clarify the impact of spermidine on the suppression capacity of Tregs in NOD mice.

In late onset NOD mice, a reduced proportion of Tregs correlated with a higher diabetes incidence and an increased proportion of blood CD8+ T-cells. A previous report has also shown that a reduced proportion of Tregs led to diabetes onset in NOD mice^41^. Similarly, spermidine reduced the proportion of Tregs in cancer but tumor growth was only diminished in the presence of CD8+ T-cells in an autophagy-dependent manner^42^. In line with reduced Tregs in spermidine-treated mice, it was shown that polyamine depletion leads to an increased number of Tregs in NOD mice^27^.

Contrasting effects of spermidine on Tregs might arise from differences in the age of diabetes onset, similar to a diverse time of onset in human type 1 diabetes^43^. Due to a different genetic predisposition, early onset mice might have a more severe pancreatic inflammation leading to increased peripheral inflammation and tolerance. Additionally, the duration of spermidine treatment or age of the animals could influence Treg number and function. Further studies investigating various treatment durations in different age groups of pre-diabetic and diabetic NOD mice might be able to identify underlying mechanisms.

In conclusion, we showed that daily oral 10 mM spermidine treatment led to a higher diabetes incidence in NOD mice by elevating peripheral inflammation and reducing the proportion of suppressive Tregs. Spermidine did not alter pancreatic inflammation and did not induce autophagy-dependent insulin degradation. Although autophagy has been shown to be crucial in maintaining beta cell function under physiological conditions and in type 2 diabetes, we showed that spermidine treatment did not reduce diabetes incidence but increased pro-inflammatory T-cells in a type 1 diabetes model. Future studies should further elucidate the role of spermidine in immune cell modulation and autophagy induction in different age groups of pre-diabetic and diabetic NOD mice.

## Supplementary Information

Supplementary Information.
