# Supplementary material for: The effect of spermidine on autoimmunity and beta cell function in NOD mice
Source: Sci Rep. 2022 Mar 16;12:4502. doi: 10.1038/s41598-022-08168-2 (PMC8927410; doi:10.1038/s41598-022-08168-2)
Supplement: Supplementary file 1 — Supplementary Information. [file 41598_2022_8168_MOESM1_ESM.docx]

**Electronic supplementary Material**

Supplementary Figure 1: (a) Body weight (BW) at the beginning of the study (ctrl n=28 and spd n=30). (b) Food consumption throughout the study, each bar graph represents 2-3 independent measurements from each of the two cages. (c) Average of blood glucose levels measured from 7 to 32 weeks of treatment. Black squares show ctrl mice and red circles show spd mice. Data is shown as mean ± SD. (a, b) Unpaired Student´s t-test and (c) two-way ANOVA with Sidak´s post-hoc test were performed to compare ctrl and spd groups.

Supplementary Figure 2: Spermidine did not change the number of insulin granules in pancreatic beta cells. (a) Representative images of fusion structures. (a1) Black arrowhead indicates mature granules, white arrowhead indicates fusion of mature granules ‑ bright surrounding halo of both granules is fused. (a2) 3 mature granules show a fusion of their surrounding membranes. (a3) Black arrows indicate fusion of transforming immature granules with mature granules, membrane of both types of granules is open. (a4) White arrow indicates cluster of immature granules – fusion of the white surrounding halo. (b) Types of fusion insulin granules normalized to filled vesicles analyzed. Scale bar 0.5 µm. Nondiabetic ctrl mice (n=3) and nondiabetic spd mice (n=3). Each datapoint represents the average of 3 islets from one mouse; area of 332 µ$m^{2}$ - 2272 µ$m^{2}$ from each islet was analyzed. Data is shown as mean ± SD. Two-way ANOVA with Sidak´s post-hoc test was used as statistical analysis.

**
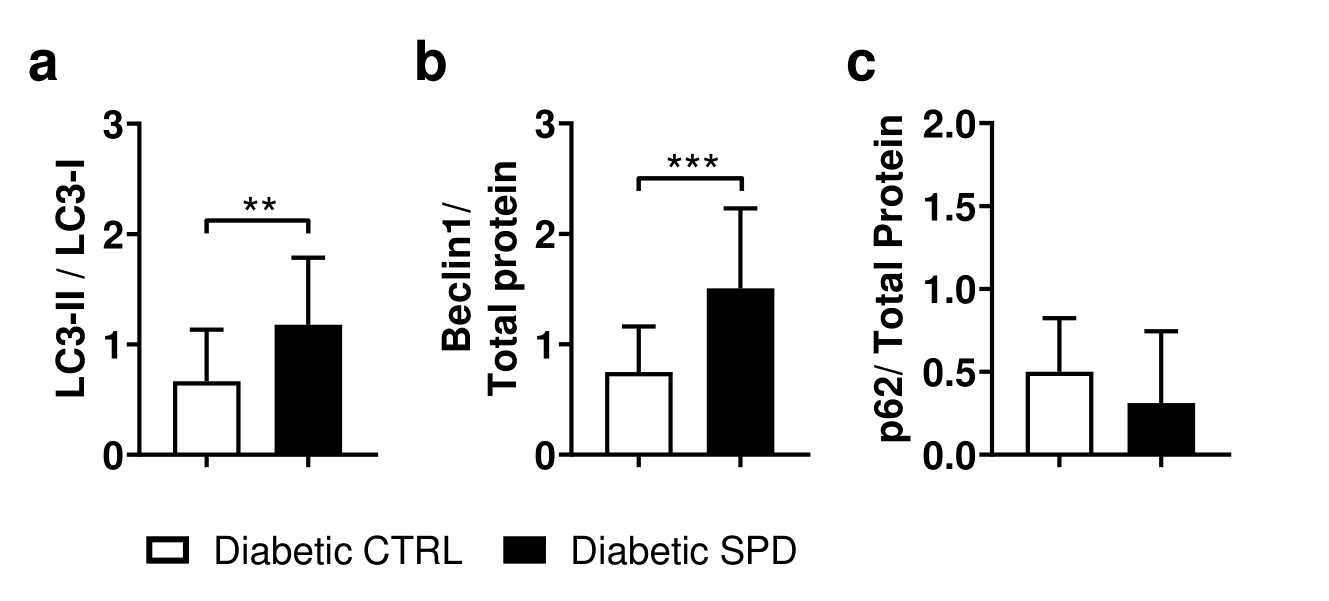
**Supplementary Figure 3: Daily oral spermidine treatment enhanced autophagy in pancreas of diabetic mice. Samples were probed for LC3, Beclin-1, p62 and stained with coomassie blue for total protein in western blot. (a) LC3-II to LC3-I ratio. (b) Beclin-1 normalized to total protein. (c) p62 normalized to total protein. White bar shows diabetic ctrl mice (n=16-17) and black bar shows diabetic spd mice (n=18-19). Data is shown as mean ± SD. Mann-Whitney U test or unpaired Student´s t-test were used as statistical analysis. *p*** < 0.01, *p**** < 0.001.

Supplementary Figure 4: Spermidine treatment did not alter ornithine and spermine levels. Pancreas, pLN, spleen, thymus, heart, plasma and whole blood samples were analyzed in male NOD mice after 4 weeks of 10 mM spermidine treatment for (a) ornithine and (b) spermine levels. Data is shown as nmol/ g and mean ± SD. White bar shows ctrl mice (n=8) and black bar shows spd mice (n=9-10). Mann-Whitney U test or unpaired Student´s t-test was used as statistical analysis.

##
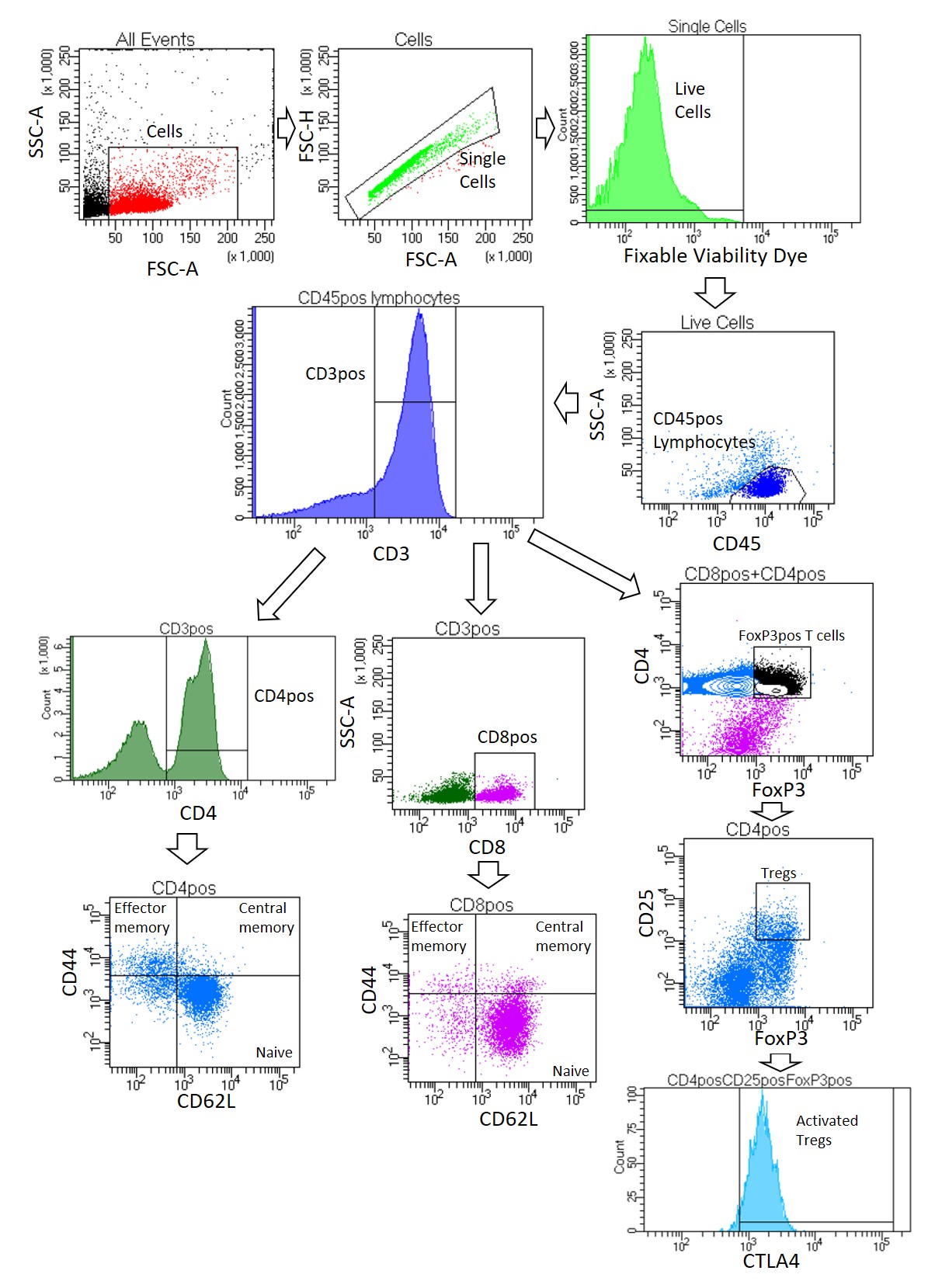


## Supplementary Figure 5: Gating strategy for naïve CD4+ T-cells, effector memory CD4+ T-cells, central memory CD4+ T-cells, naïve CD8+ T-cells, effector memory CD8+ T-cells, central memory CD8+ T-cells, FoxP3+ T-cells, Tregs and activated Tregs. All gates were set according to FMO controls (Fluorochrome-Minus-One Controls) and isotype control stainings. All immune cell populations were always gated for singularity (FSC-H vs FSC-A) and viability. Then T cell populations were gated accordingly: CD4+ T-cells (CD45+, CD3+, CD4+), CD8+ T-cells (CD45+, CD3+, CD8+), naïve CD4+ T-cells (CD45+, CD3+, CD4+, CD44(-), CD62L+), effector memory CD4+ T‑cells (CD45+, CD3+, CD4+, CD44+, CD62L(-)), central memory CD4+ T-cells (CD45+, CD3+, CD4+, CD44+, CD62L+), naïve CD8+ T-cells (CD45+, CD3+, CD8+, CD44(-), CD62L+), effector memory CD8+ T-cells (CD45+, CD3+, CD8+, CD44+, CD62L(-)), central memory CD8+ T-cells (CD45+, CD3+, CD8+, CD44+, CD62L+), FoxP3+ T‑cells (CD45+, CD3+, CD4+, FoxP3+), regulatory T-cells (CD45+, CD3+, CD4+, FoxP3+, CD25+), activated regulatory T-cells (CD45+, CD3+, CD4+, FoxP3+, CD25+, CTLA4+).

**
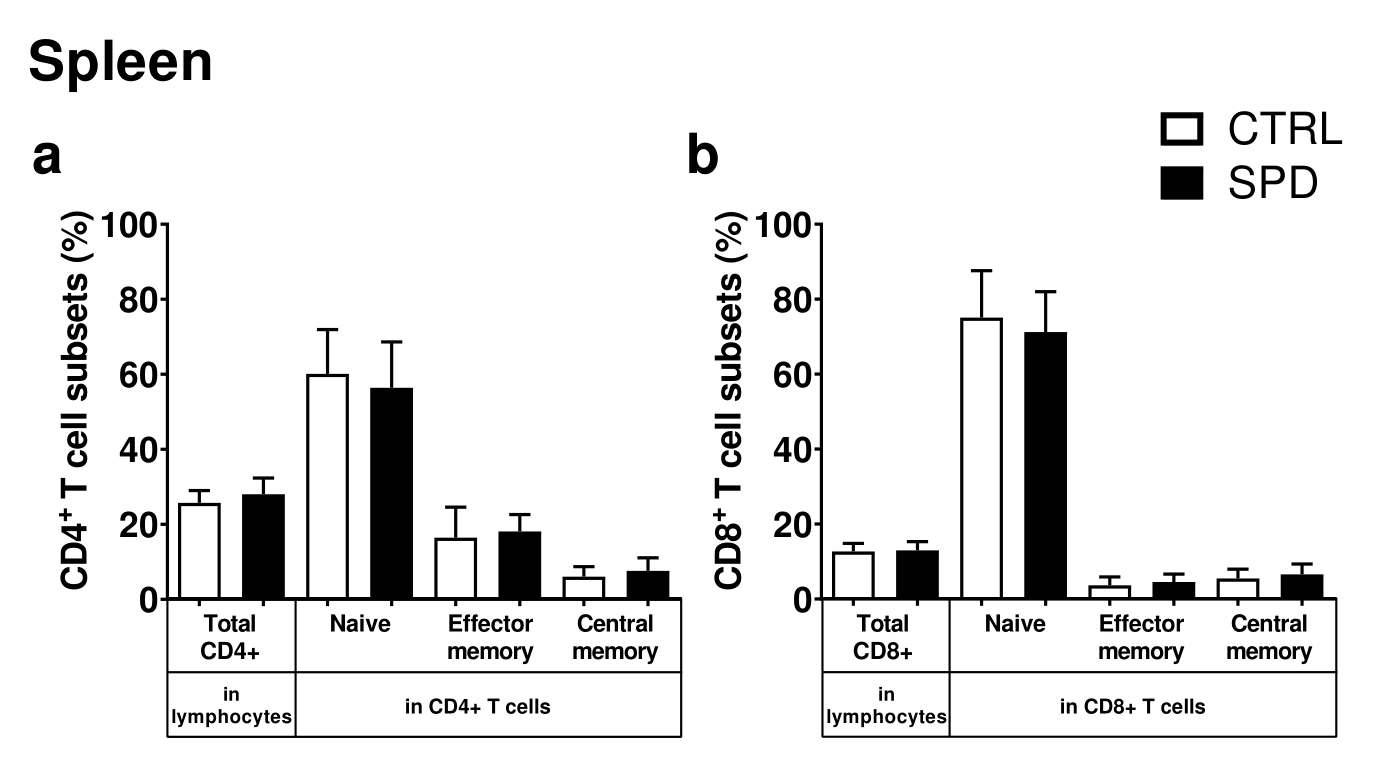
**Supplementary Figure 6: Spermidine did not alter CD4+ T-cells and CD8+ T-cells in spleen of diabetic mice. (a) Total CD4+ T-cells, naïve CD4+ T-cells, effector memory CD4+ T-cells and central memory CD4+ T-cells and (b) total CD8+ T-cells, naïve CD8+ T-cells, effector memory CD8+ T-cells and central memory CD8+ T-cells were examined in spleen. White bar shows diabetic ctrl mice (n=16) and black bar shows diabetic spd mice (n=24). Data is shown as mean ± SD. Mann-Whitney U test or unpaired Student´s t-test were used as statistical analysis.

**
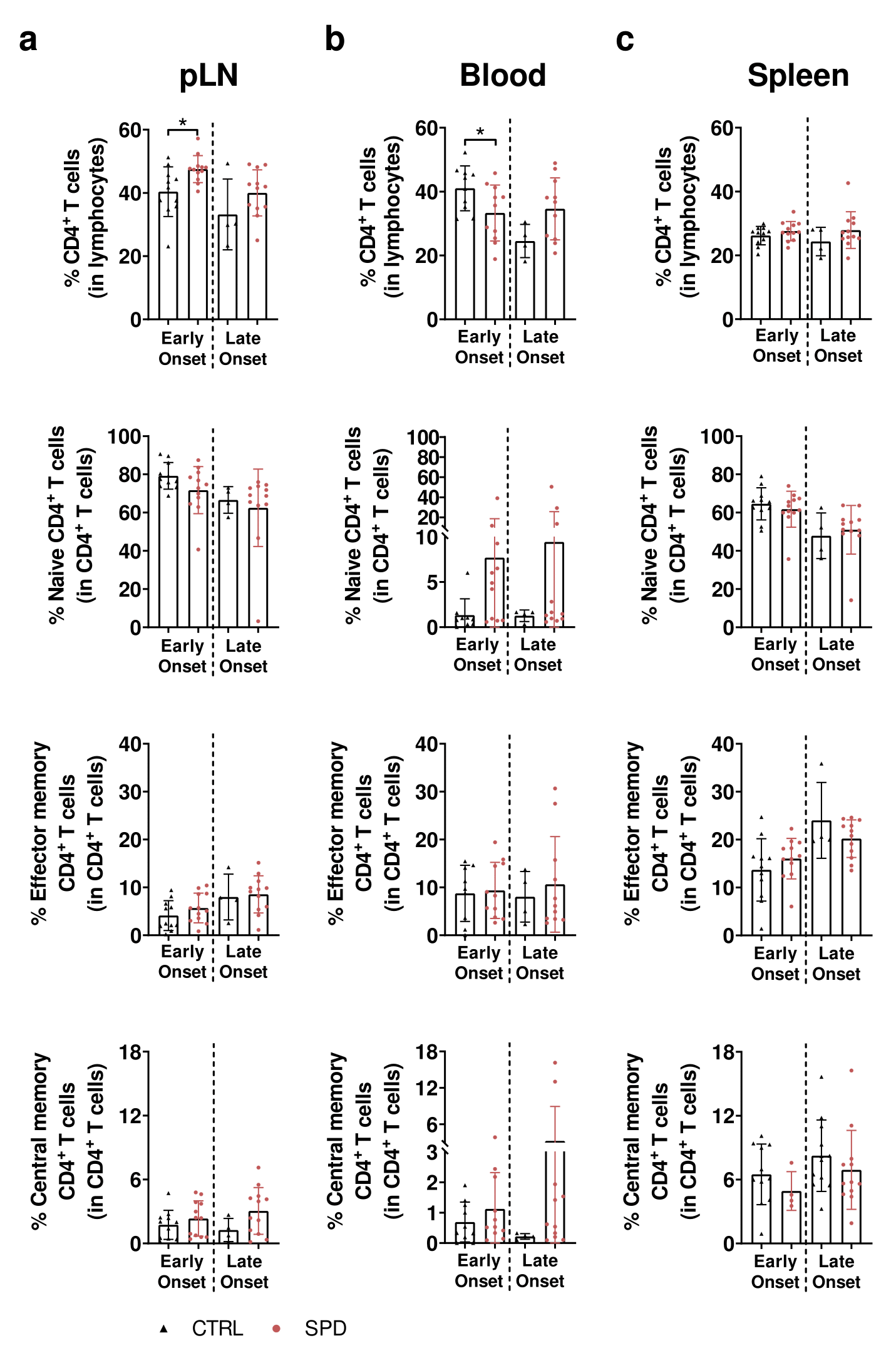
**

Supplementary Figure 7: The effect of spermidine treatment on CD4+ T-cells subsets in early and late onset mice. (a) pLN. (B) Blood. (c) Spleen. Total CD4+ T-cells, naïve CD4+ T-cells, effector memory CD4+ T-cells and central memory CD4+ T-cells were analyzed. Data is shown as mean ± SD. Diabetic mice were grouped as early onset (< 21 weeks of age) and late onset (≥ 21 weeks of age). Early onset ctrl mice (n=10‑11), early onset spd mice (n=11‑12), late onset ctrl mice (n=4-5), late onset spd mice (n=11‑12). Black triangles show ctrl mice and red circles show spd mice. Mann-Whitney U test or unpaired Student´s t-test was used as statistical analysis. *p** < 0.05.

**
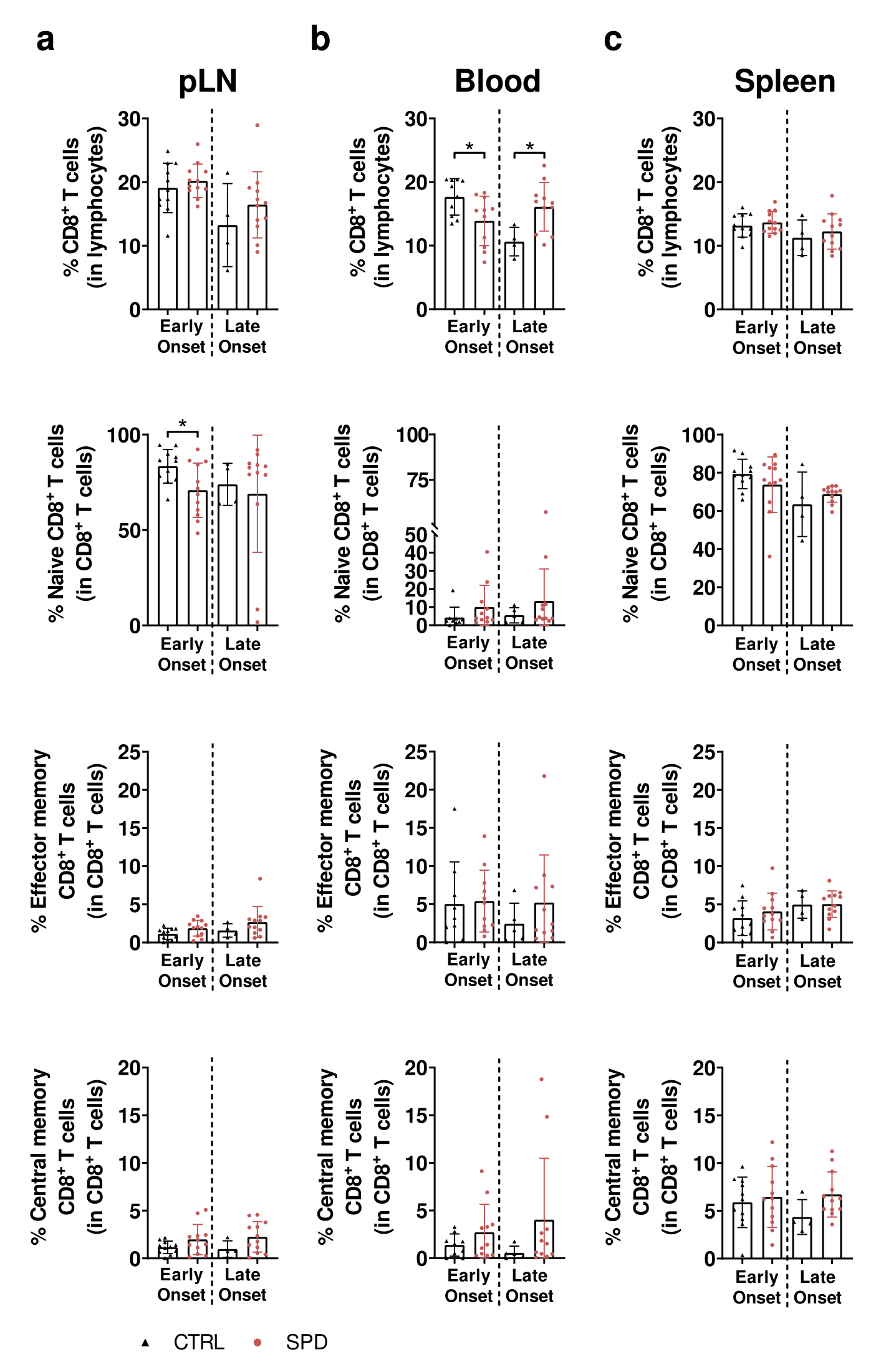
**

Supplementary Figure 8: The effect of spermidine treatment on CD8+ T-cells subsets in early and late onset mice. (a) pLN. (B) Blood. (c) Spleen. Total CD8+ T-cells, naïve CD8+ T-cells, effector memory CD8+ T-cells and central memory CD8+ T-cells were analyzed. Data is shown as mean ± SD. Diabetic mice were grouped as early onset (< 21 weeks of age) and late onset (≥ 21 weeks of age). Early onset ctrl mice (n=10-11), early onset spd mice (n=11‑12), late onset ctrl mice (n=4-5), late onset spd mice (n=11‑12). Black triangles show ctrl mice and red circles show spd mice. Mann-Whitney U test or unpaired Student´s t-test was used as statistical analysis. *p** < 0.05.

**
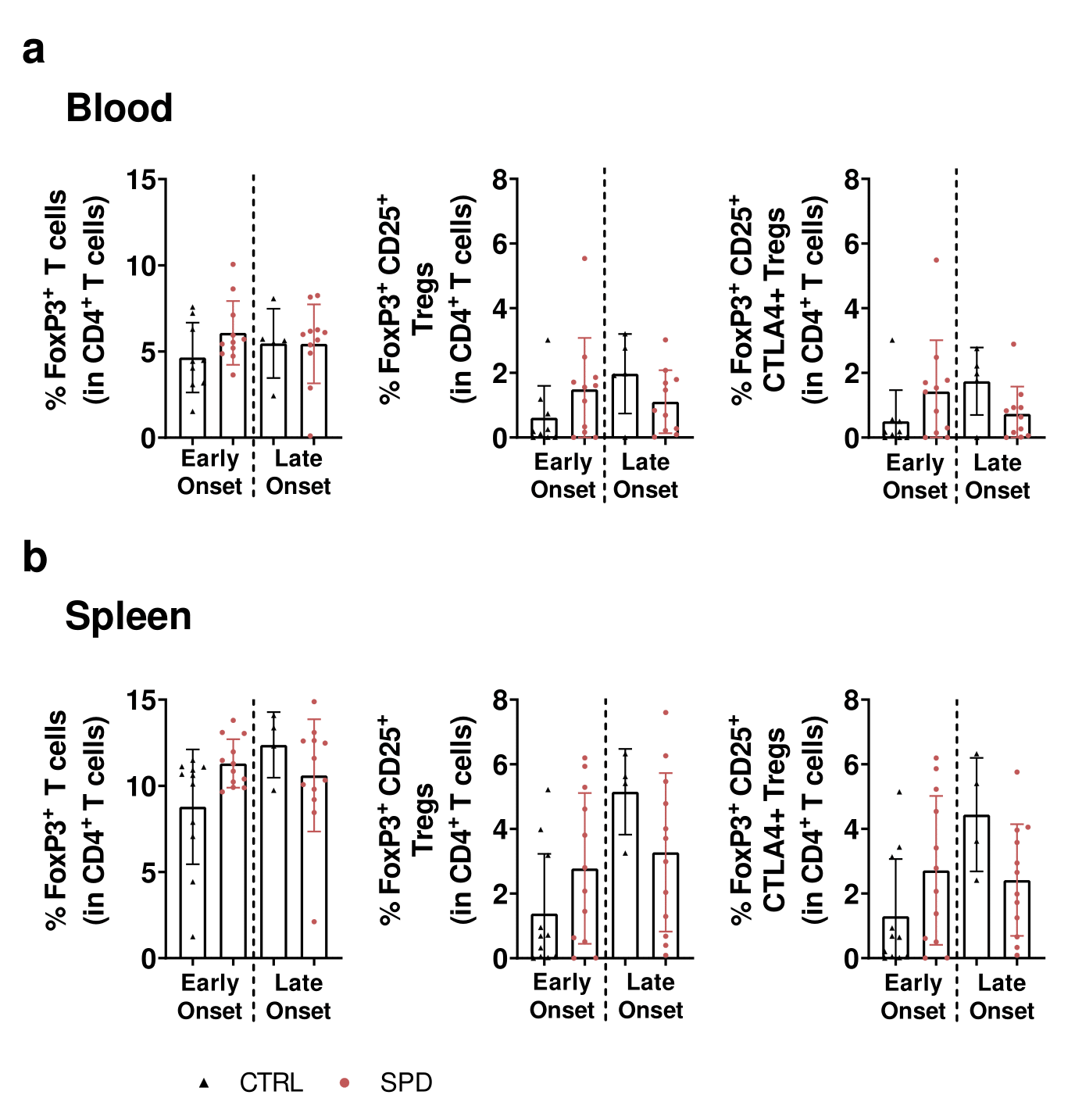
**

Supplementary Figure 9: The effect of spermidine treatment on FoxP3+ T-cells and Treg subsets in early and late onset mice. (a) Blood. (b) Spleen. Diabetic mice were grouped as early onset (< 21 weeks of age) and late onset (≥ 21 weeks of age). Early onset ctrl mice (n=11), early onset spd mice (n=12), late onset ctrl mice (n=4), late onset spd mice (n=12). Black triangles show ctrl mice and red circles show spd mice. Data is shown as mean ± SD. Mann‑Whitney U test or unpaired Student´s t test was used as statistical analysis to compare ctrl and spd mice.


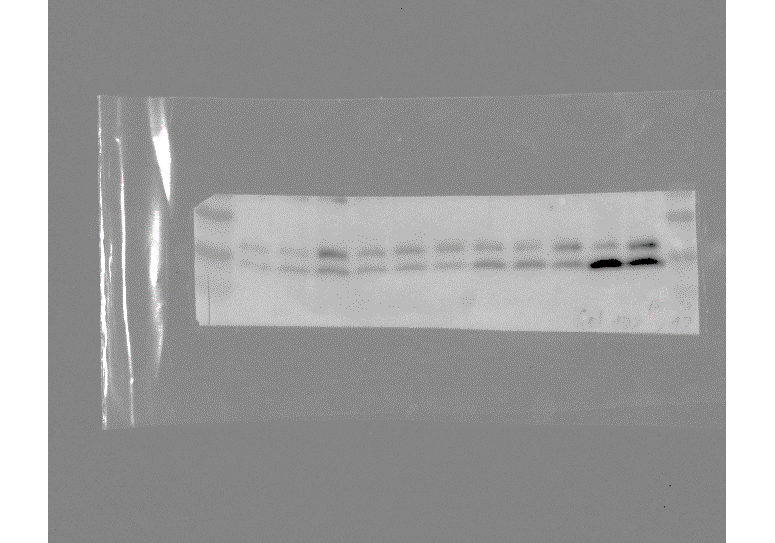


20

15

10

25

✄

LC3-I (16 kDa)

LC3-II (14 kDa)

✄


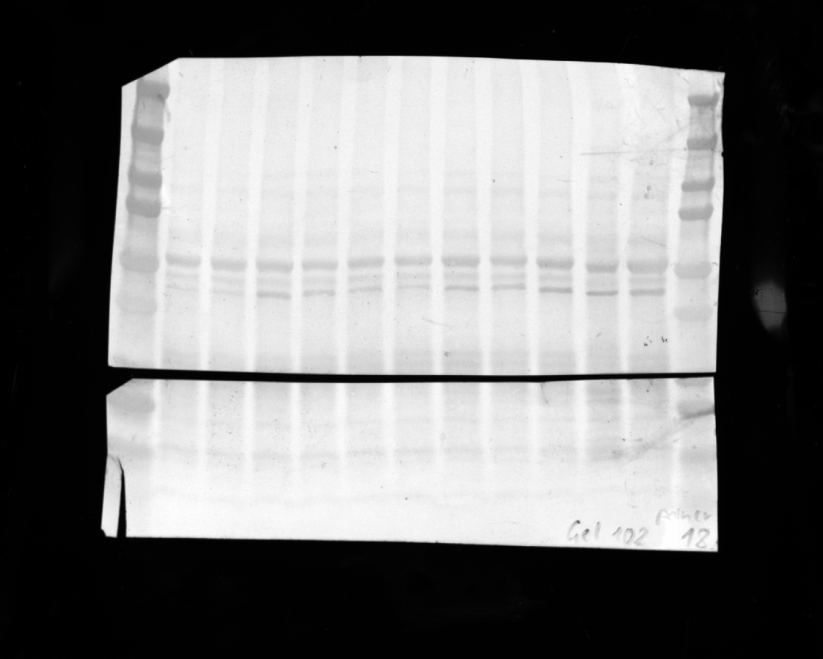


250

50

150

100

75

37

25

20

15

10

✄

✄

Commassie Staining

(50 kDa)

Early onset diabetic

ctrl mice

Nondiabetic

ctrl mice

Positive Ctrl

Pooled sample

Full images for extended data Fig. 4b


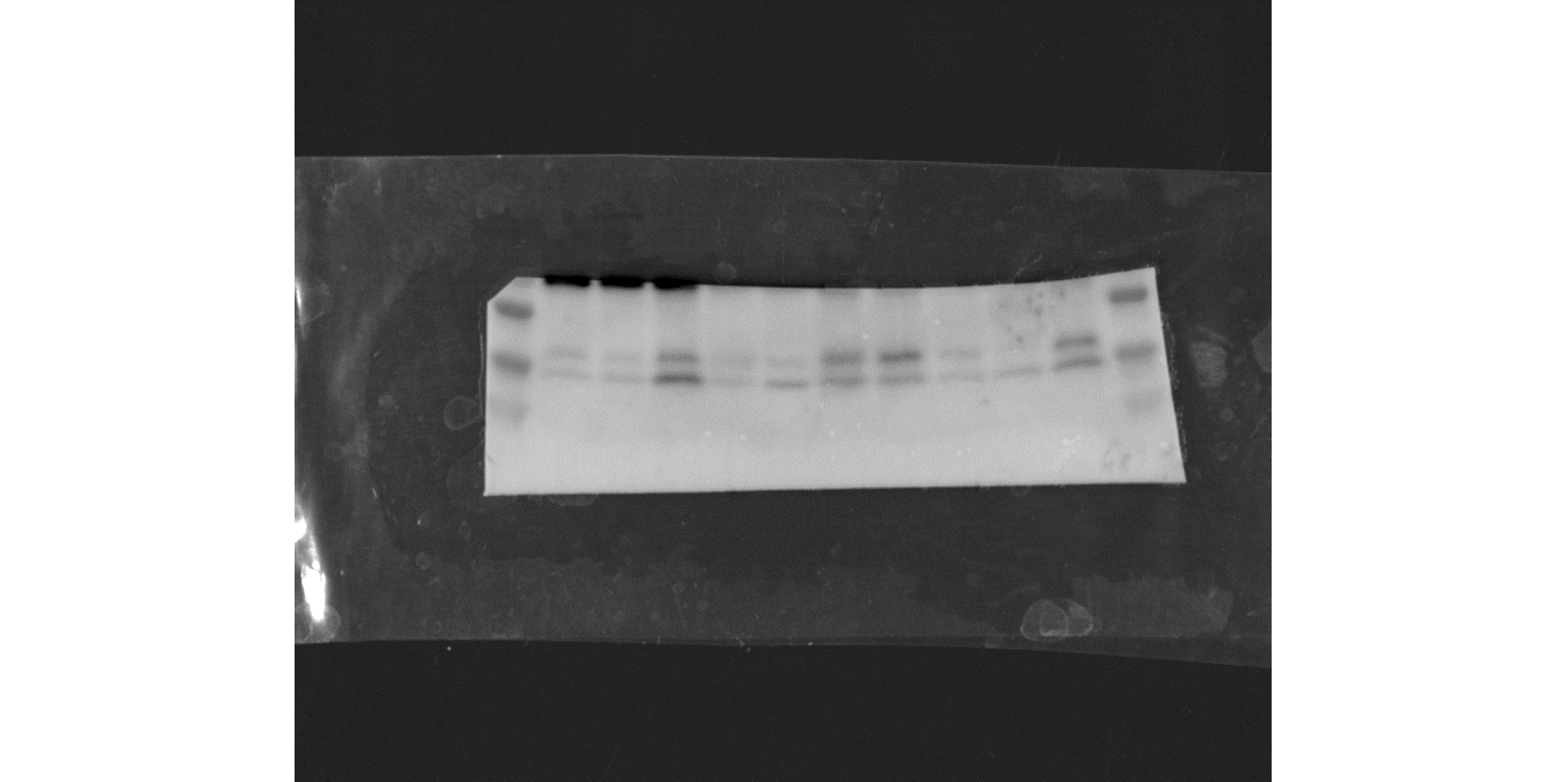


LC3-I (16 kDa)

LC3-II (14 kDa)

20

15

10

25

✄

✄

Comassie Staining

(50 kDa)


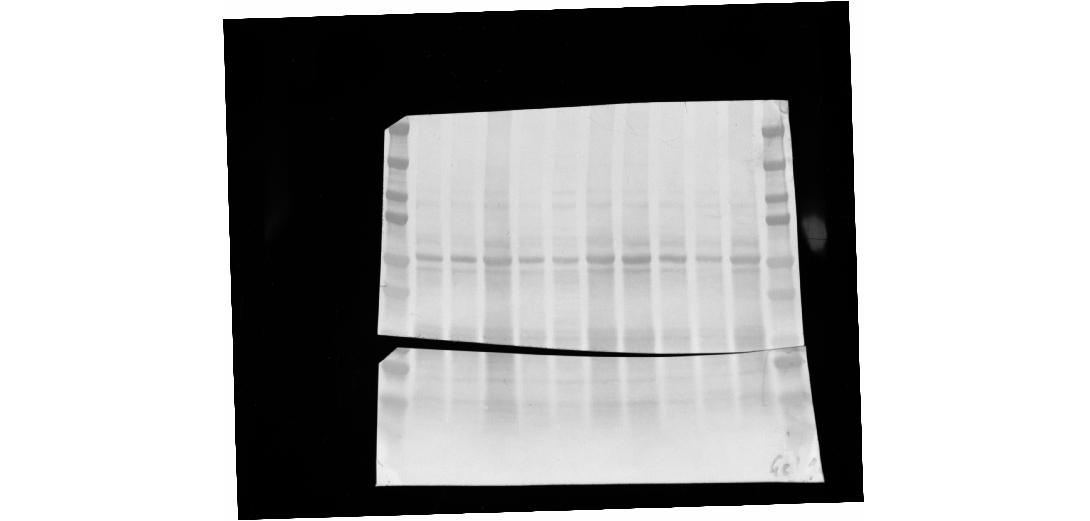


250

50

150

100

75

37

25

20

15

10

✄

✄

Early onset diabetic

spd mice

Nondiabetic

spd mice

Positive Ctrl

Pooled sample

Full images for extended data Fig. 4b

**b**

**a**

Figure continues on the next page

**d**

**c**


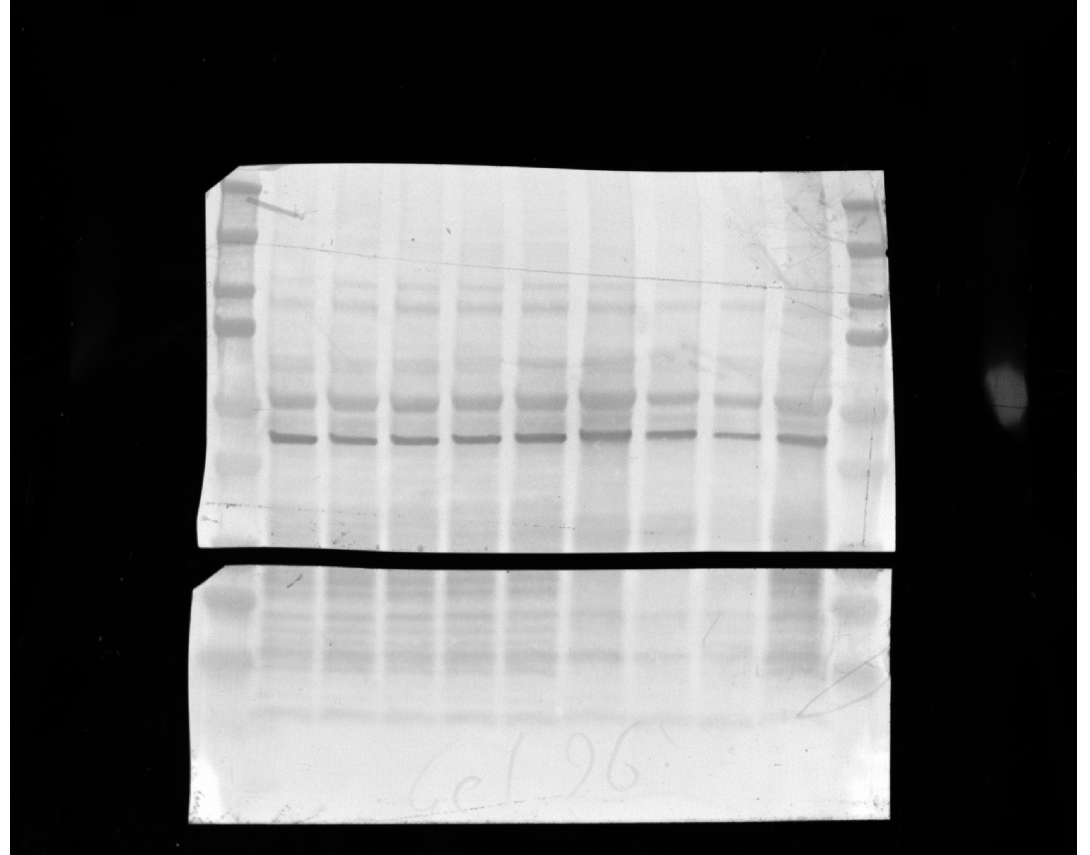


Comassie Staining

(50 kDa)

250

50

150

100

75

37

25

✄

✄

20

15

10

LC3-I (16 kDa)

LC3-II (14 kDa)


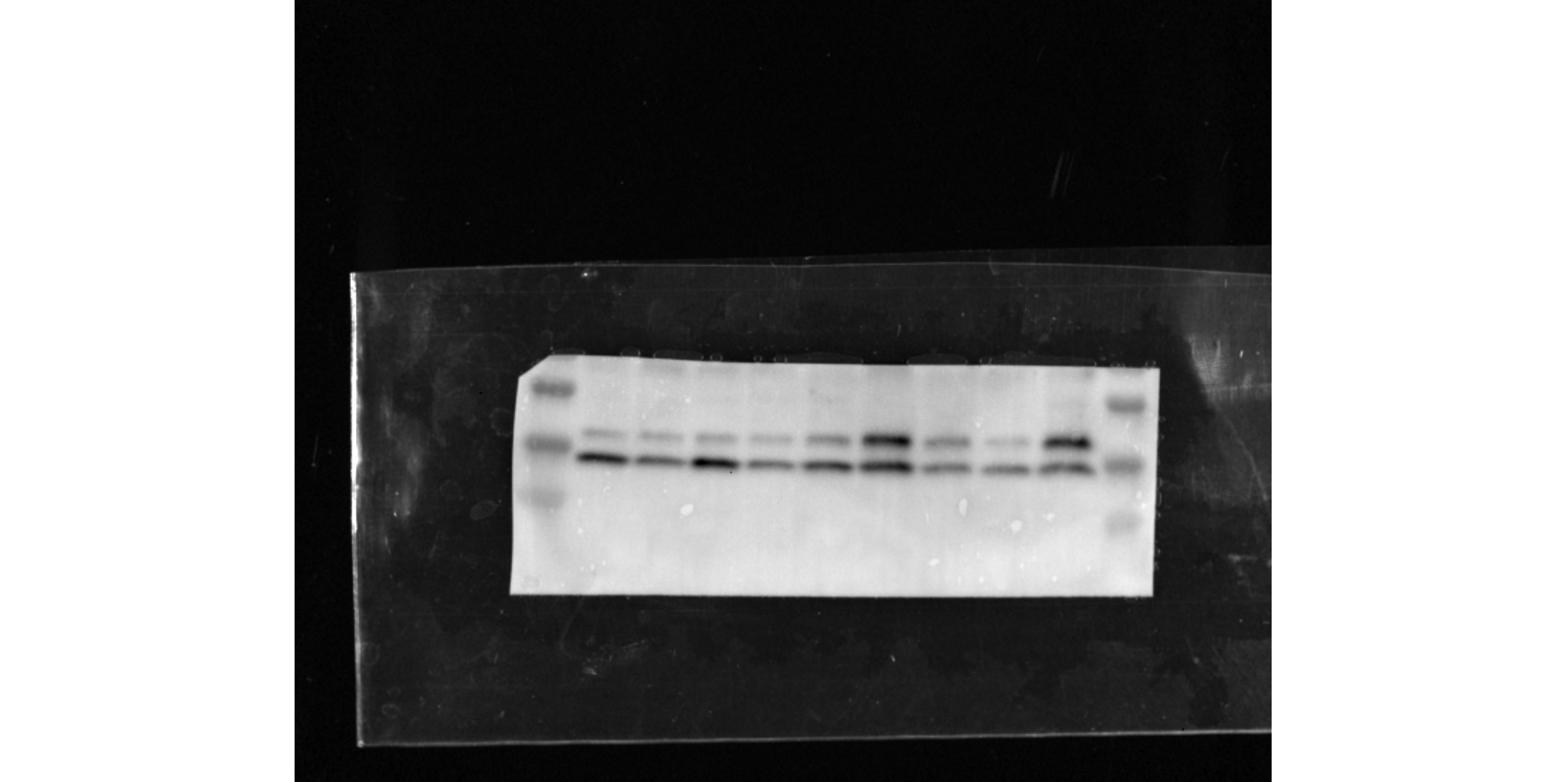


20

15

10

25

✄

✄

Late onset diabetic

ctrl mice

Nondiabetic

ctrl mice

Positive Ctrl

Pooled sample

Full images for extended data Fig. 4b


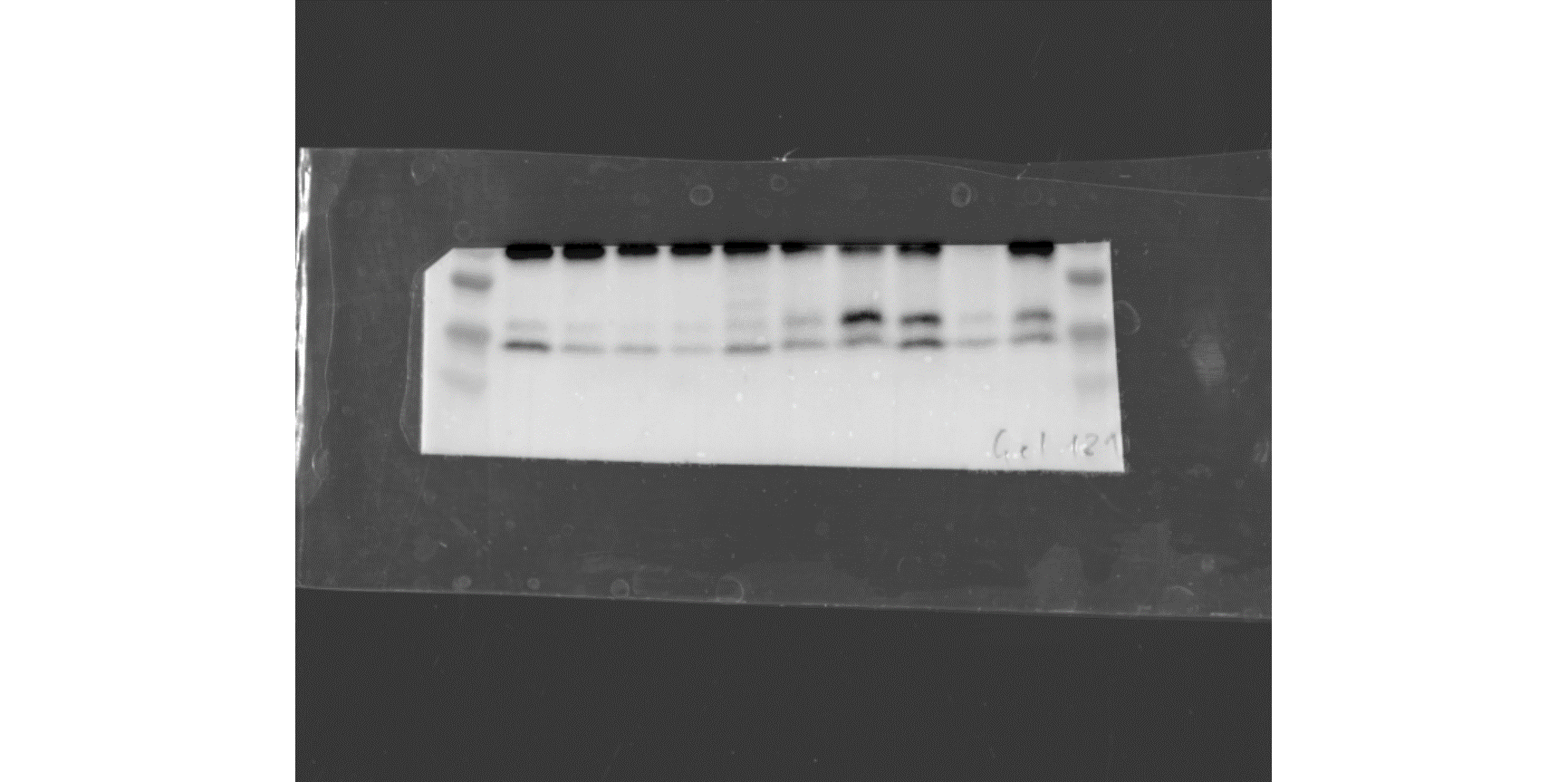


LC3-I (16 kDa)

LC3-II (14 kDa)

20

15

10

25

✄

✄


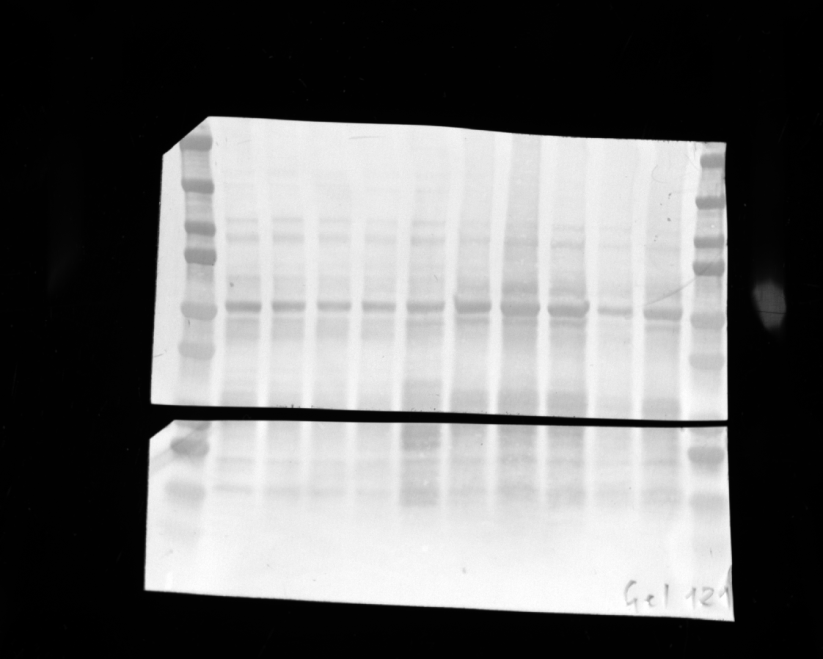


✄

250

50

150

100

75

37

25

20

15

10

✄

Comassie Staining

(50 kDa)

Late onset diabetic

spd mice

Nondiabetic

spd mice

Positive Ctrl

Pooled sample

Full images for extended data Fig. 4b

Figure continues on the next page

**e**

**f**


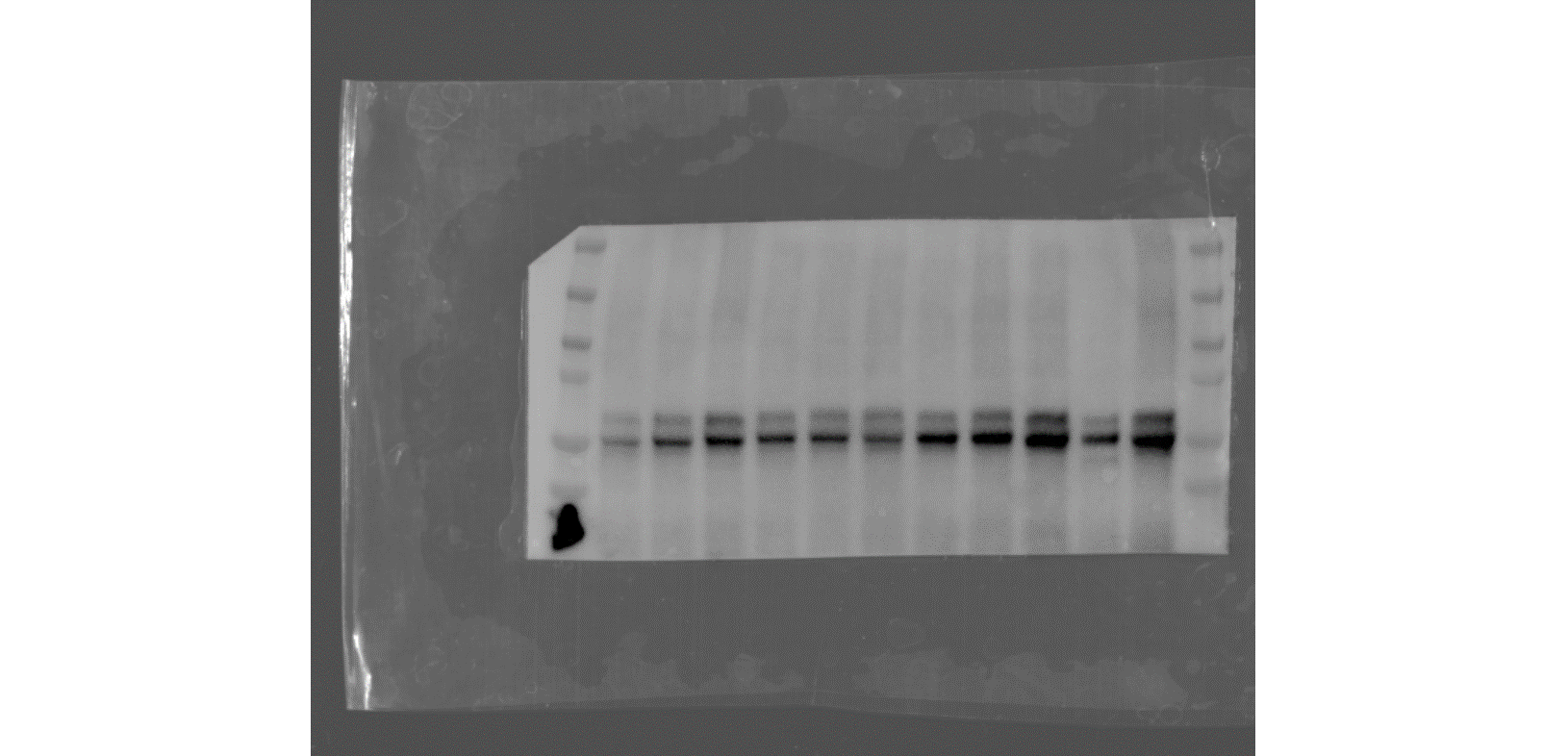


Beclin1 (60 kDa)

250

50

150

100

75

37

25

✄

✄

p62 (62 kDa)


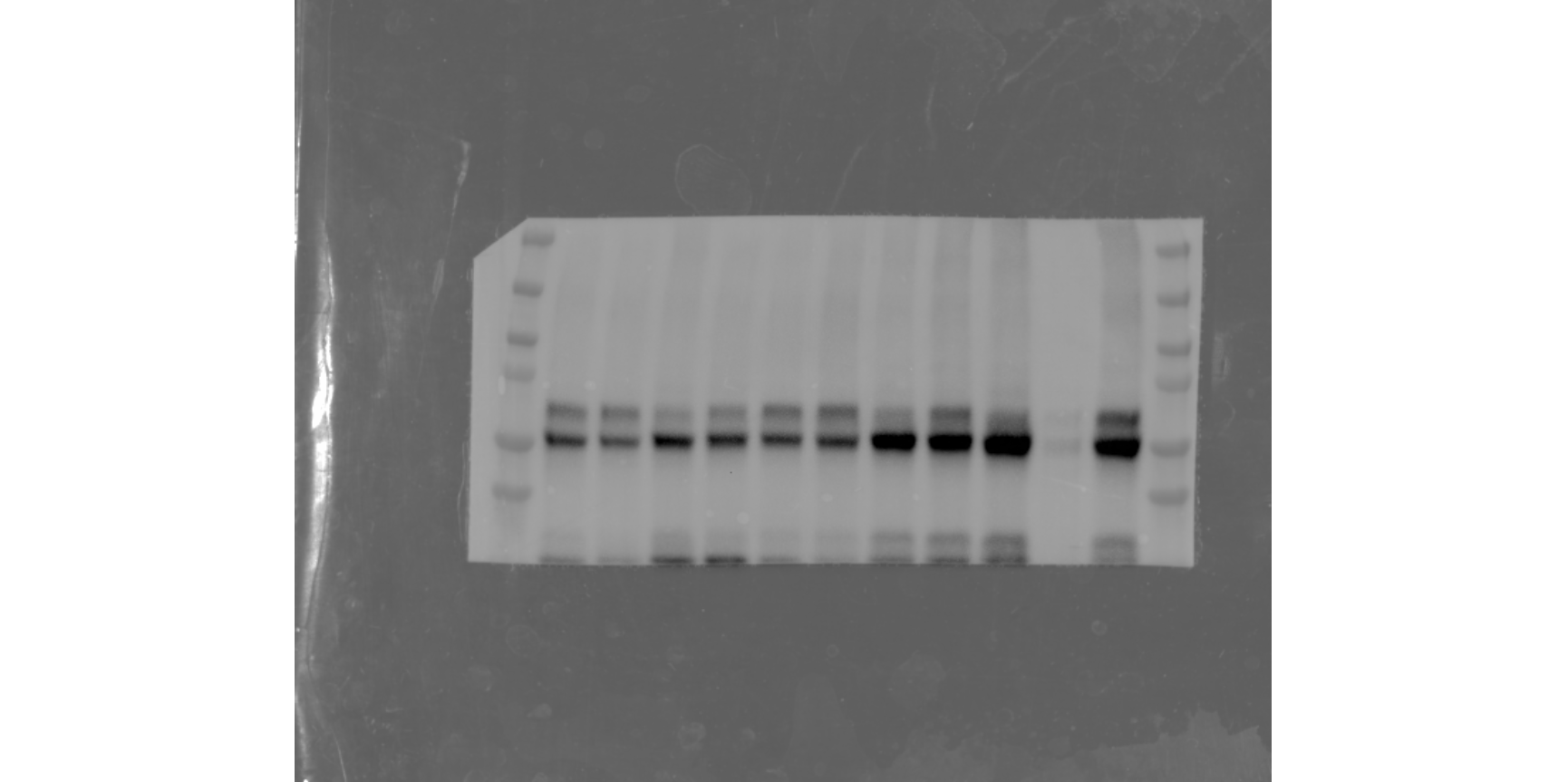


250

50

150

100

75

37

25

✄

✄


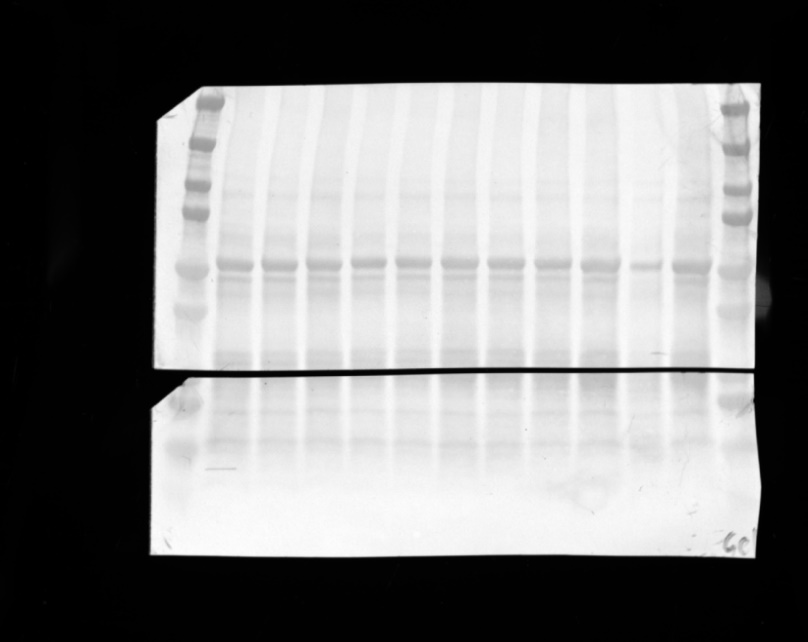


Comassie Staining

(50 kDa)

250

50

150

100

75

37

25

20

15

10

✄

✄

Early onset diabetic

ctrl mice

Nondiabetic

ctrl mice

Positive Ctrl

Pooled sample

Full images for extended data Fig. 4b

Beclin1 (60 kDa)


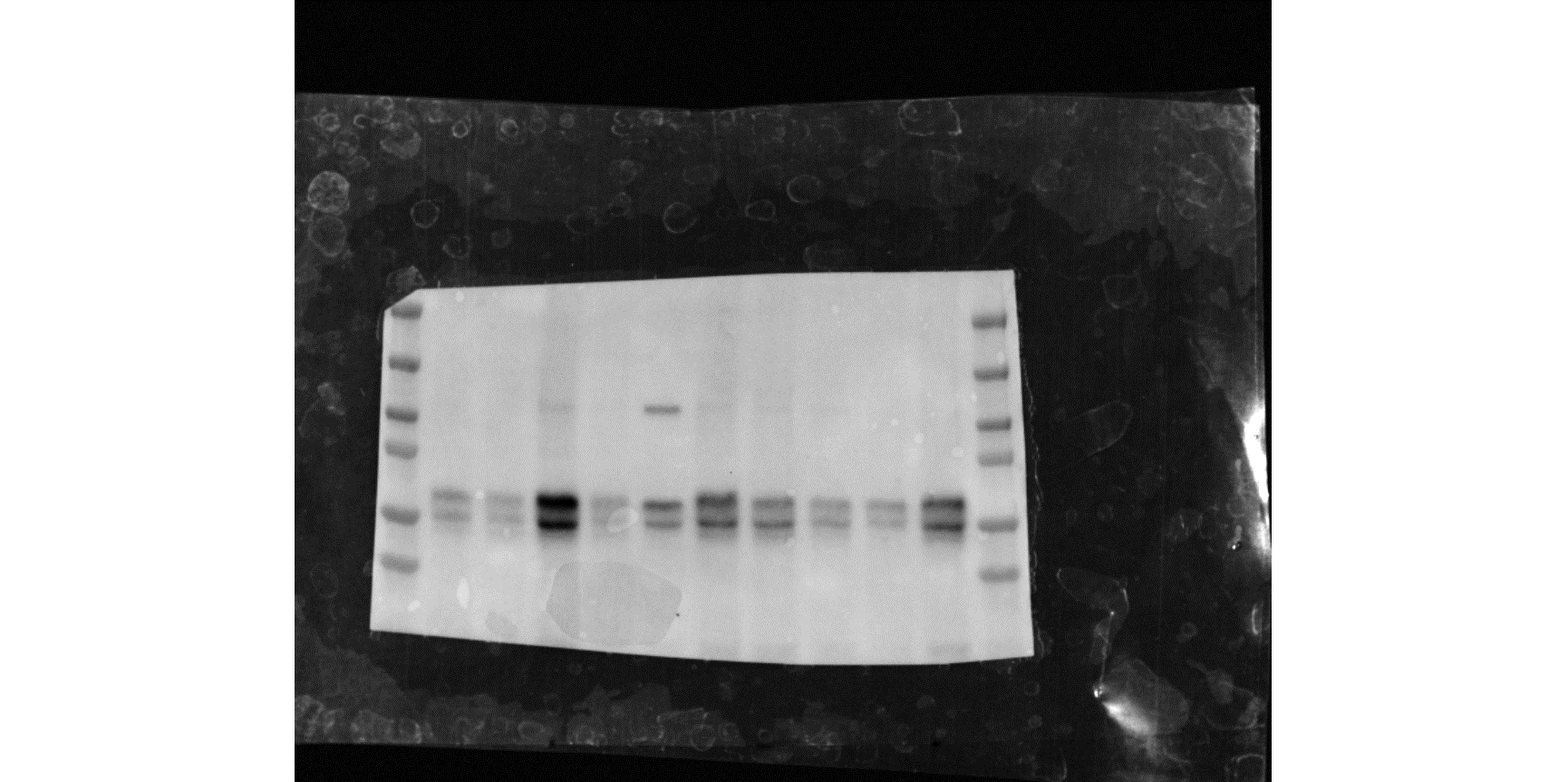


250

50

150

100

75

37

25

✄

✄


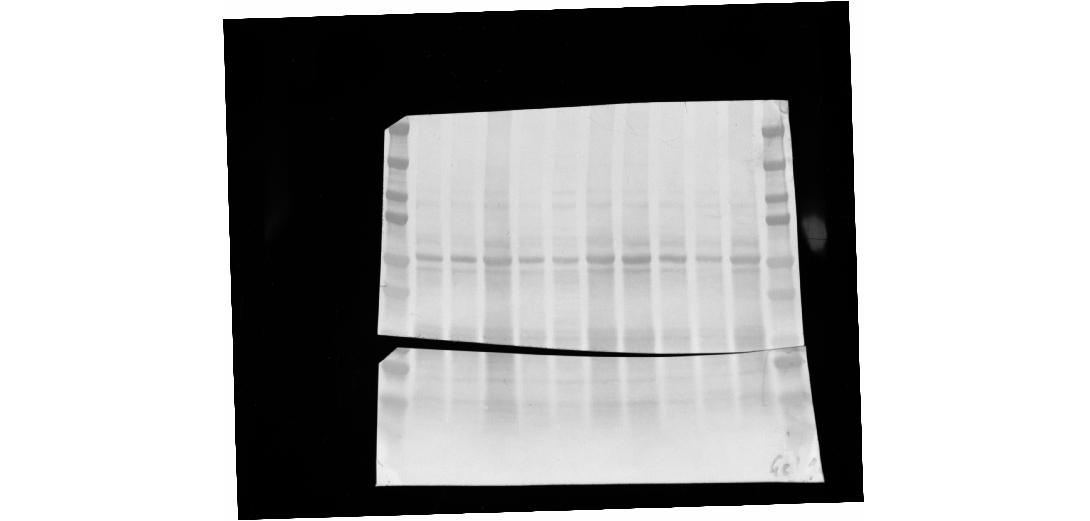


250

50

150

100

75

37

25

20

15

10

✄

✄


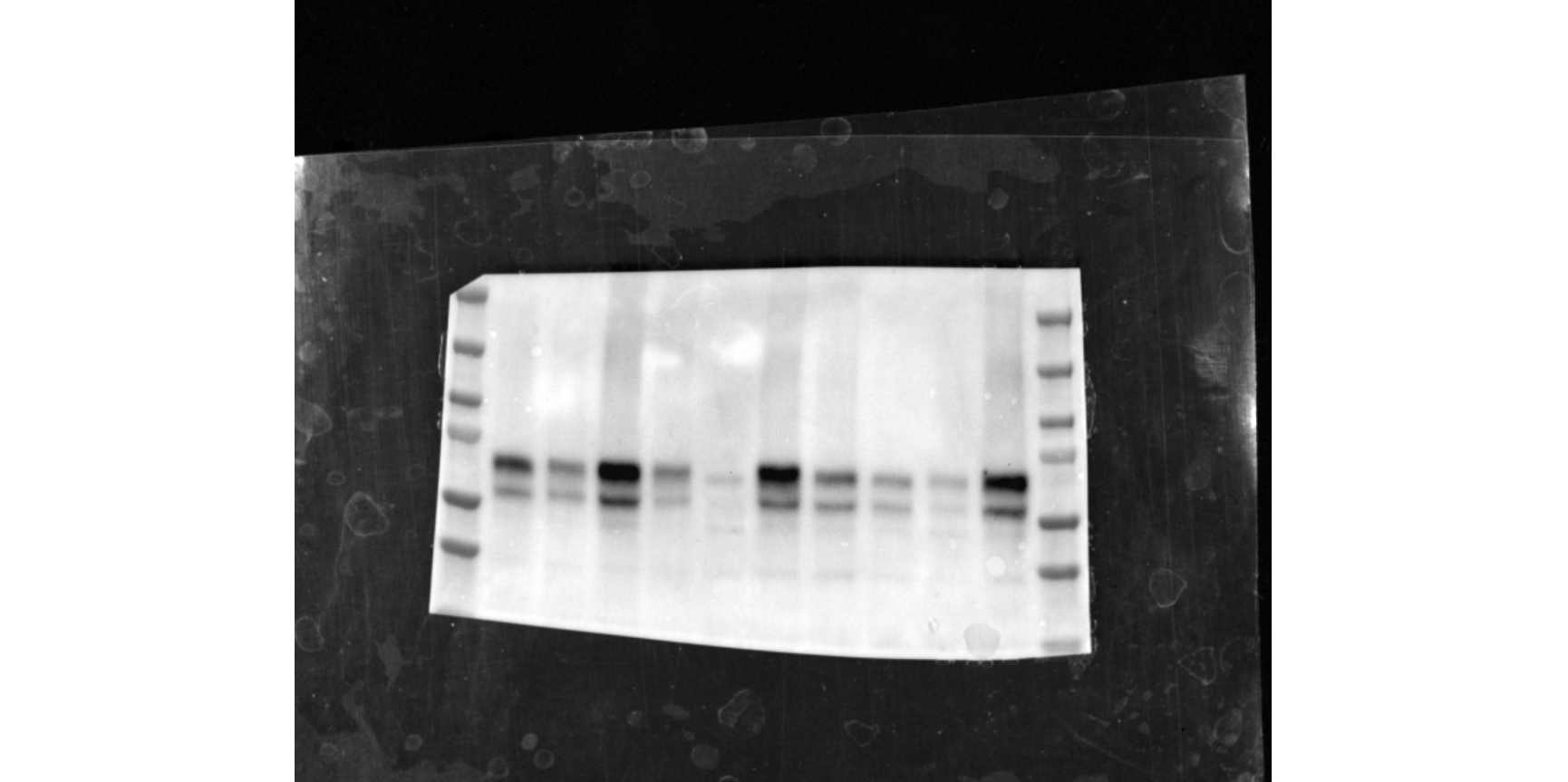


p62 (62 kDa)

✄

✄

250

50

150

100

75

37

25

Early onset diabetic

spd mice

Nondiabetic

spd mice

Positive Ctrl

Pooled sample

Full images for extended data Fig. 4b

Comassie Staining

(50 kDa)

Figure continues on the next page

**h**

Positive Ctrl

Pooled sample

Full images for extended data Fig. 4b

Beclin1 (60 kDa)


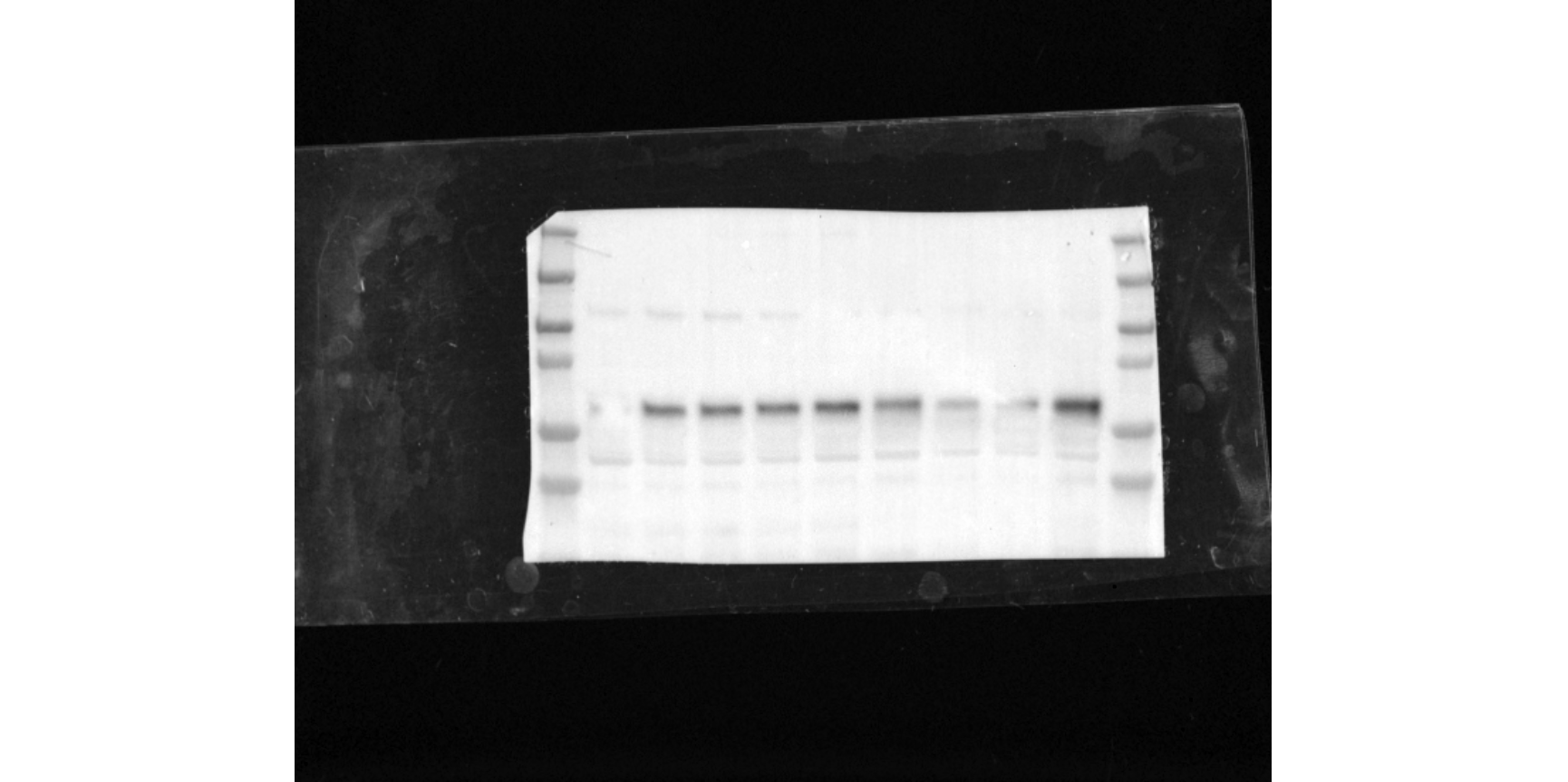


✄

250

50

150

100

75

37

25

✄


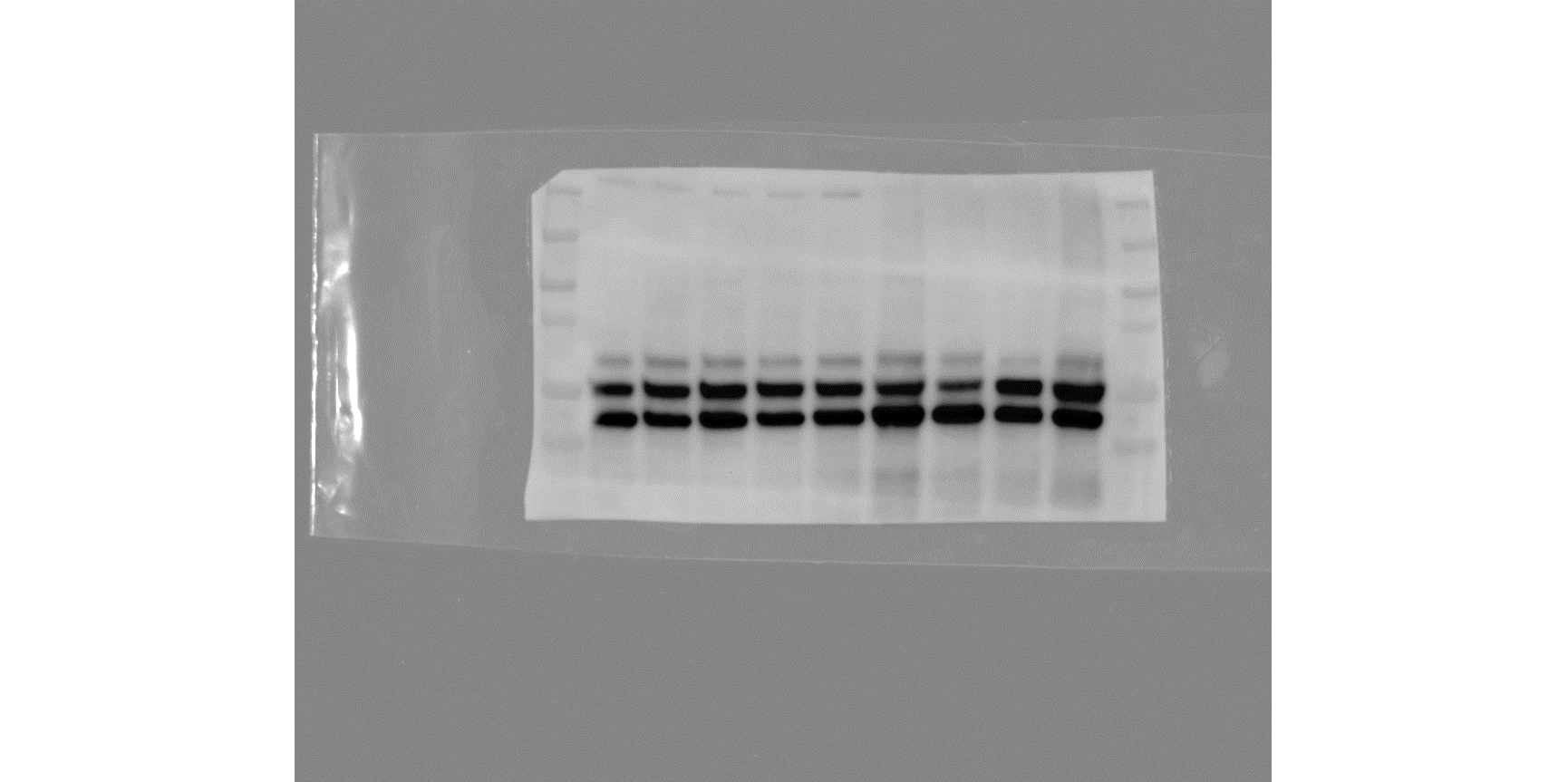


p62 (62 kDa)

250

50

150

100

75

37

25

✄

✄


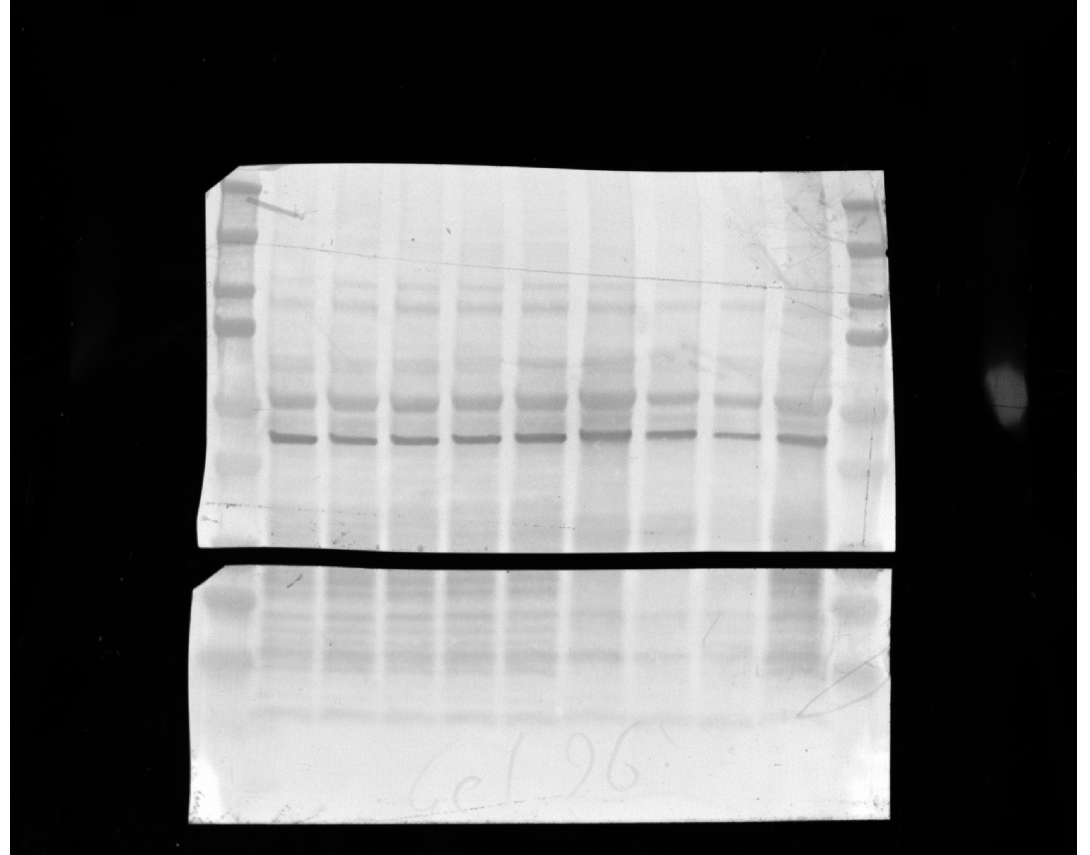


Comassie Staining

(50 kDa)

250

50

150

100

75

37

25

✄

✄

20

15

10

Late onset diabetic

ctrl mice

Nondiabetic

ctrl mice

Positive Ctrl

Pooled sample

Full images for extended data Fig. 4b


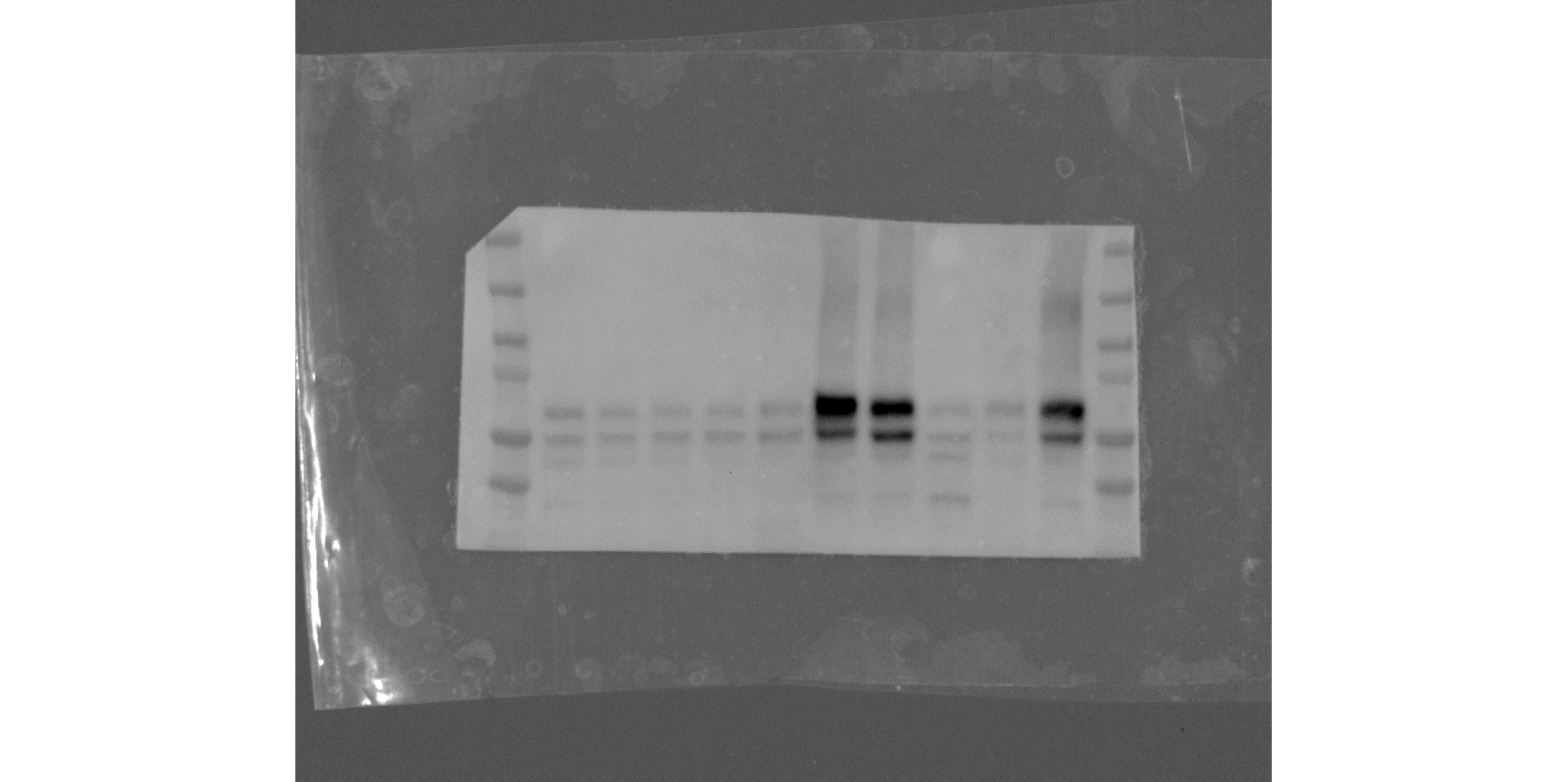

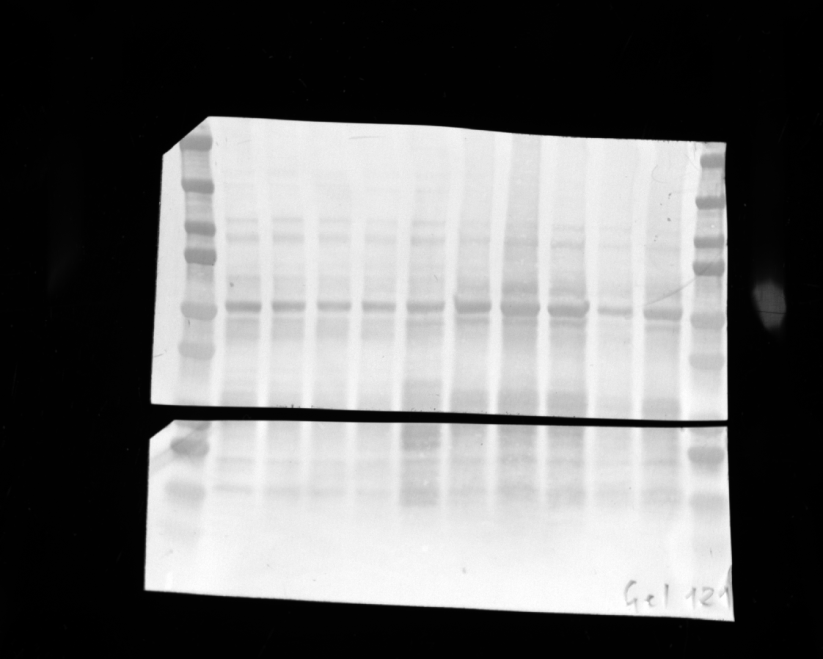


✄

✄

250

50

150

100

75

37

25

Beclin1 (60 kDa)

250

50

150

100

75

37

25

✄

✄

p62 (62 kDa)

✄

250

50

150

100

75

37

25

20

15

10

✄

Comassie Staining

(50 kDa)


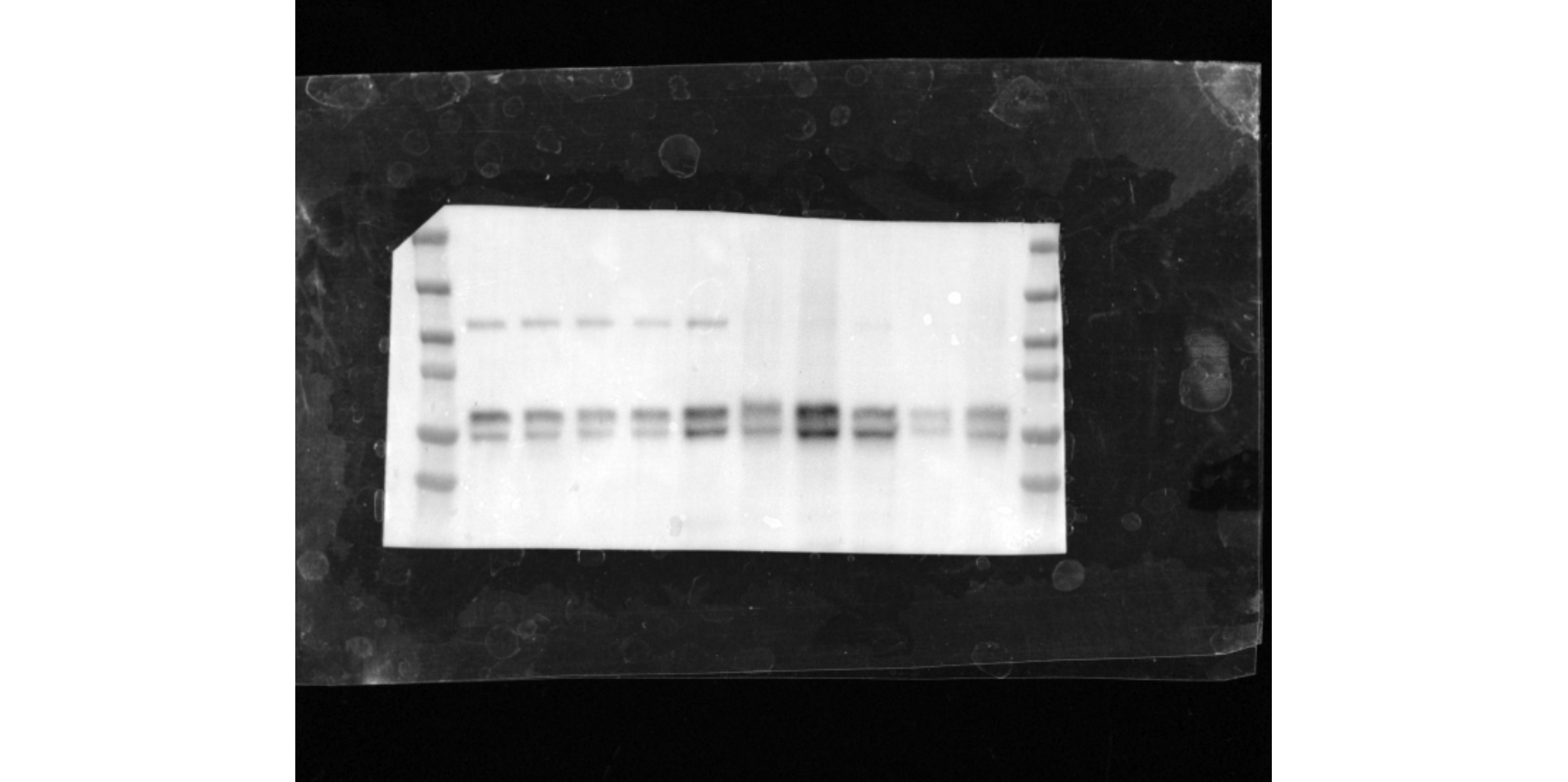


Late onset diabetic

spd mice

Nondiabetic

spd mice

**g**

Supplementary Figure 10: Full images of immunoblots presented as cropped images in Figure 4b. Samples from (a, e) early onset ctrl, (b, f) early onset spd, (c, g) late onset ctrl, (d, h) late onset spd mice.
